# Marek’s Disease Virus Regulates the Ubiquitylome of Chicken CD4^+^ T Cells to Promote Tumorigenesis

**DOI:** 10.3390/ijms20092089

**Published:** 2019-04-28

**Authors:** Xiaolu Zhou, Shanli Wu, Hongda Zhou, Mengyun Wang, Menghan Wang, Yan Lü, Zhongyi Cheng, Jiacui Xu, Yongxing Ai

**Affiliations:** 1 College of Animal Science, Jilin University, 5333 Xi An Road, Changchun 130062, Jilin, China; xiaolu_zhou0416@163.com (X.Z.); hongdazhou521@163.com (H.Z.); mengyun_wang1124@163.com (M.W.); menghanwang927@163.com (M.W.); lvyanty@jlu.edu.cn (Y.L.); jcxu@jlu.edu.cn (J.X.); 2 College of Basic Medical Sciences, Jilin University, 126 Xin Min Avenue, Changchun 130021, Jilin, China; slwu@jlu.edu.cn; 3 Jingjie PTM Biolabs Co. Ltd., 452 6th Street, Hangzhou Eco. & Tech. Developmental Area, Hangzhou 310018, Zhejiang, China; zhongyi_cheng@ptm-biolab.com

**Keywords:** Marek’s disease virus, ubiquitylome, T cell, mass spectrometry

## Abstract

Ubiquitination and deubiquitination of cellular proteins are reciprocal reactions catalyzed by ubiquitination-related enzymes and deubiquitinase (DUB) which regulate almost all cellular processes. Marek’s disease virus (MDV) encodes a viral DUB that plays an important role in the MDV pathogenicity. Chicken CD4^+^ T-cell lymphoma induced by MDV is a key contributor to multiple visceral tumors and immunosuppression of chickens with Marek’s disease (MD). However, alterations in the ubiquitylome of MDV-induced T lymphoma cells are still unclear. In this study, a specific antibody against K-ε-GG was used to isolate ubiquitinated peptides from CD4^+^ T cells and MD T lymphoma cells. Mass spectrometry was used to compare and analyze alterations in the ubiquitylome. Our results showed that the ubiquitination of 717 and 778 proteins was significantly up- and downregulated, respectively, in T lymphoma cells. MDV up- and downregulated ubiquitination of a similar percentage of proteins. The ubiquitination of transferases, especially serine/threonine kinases, was the main regulatory target of MDV. Compared with CD4^+^ T cells of the control group, MDV mainly altered the ubiquitylome associated with the signal transduction, immune system, cancer, and infectious disease pathways in T lymphoma cells. In these pathways, the ubiquitination of CDK1, IL-18, PRKCB, ETV6, and EST1 proteins was significantly up- or downregulated as shown by immunoblotting. The current study revealed that the MDV infection could exert a significant influence on the ubiquitylome of CD4^+^ T cells.

## 1. Introduction

Marek’s disease (MD) is an avian oncogenic lymphoproliferative infectious disease induced by the serotype 1 Marek’s disease virus (MDV-1) [1]. The high morbidity and mortality of MD can result in a huge economic loss in flocks worldwide [2]. The other two MDV serotypes, serotype 2 (e.g., SB-1 strain) and serotype 3 (e.g., *Turkey herpesvirus*, HVT strain), are nononcogenic viruses that are typically developed as vaccines against MD. An MD-induced tumor was the first viral tumor successfully controlled by vaccination [3], and the CVI-988 vaccine, an attenuated MDV serotype 1 strain, is the most effective vaccine against MD in poultry. Although this vaccine can prevent MD tumors and paralysis, it cannot protect birds from infection or prevent MDV shedding [2]. However, MDV is becoming increasingly virulent due to decades of the MD vaccine exposure, and strains that are able to overcome the vaccine defense are a serious threat to poultry worldwide.

MDV infects an array of immune cells. T cells, especially CD4^+^ T cells, are susceptible to infection and transformation by MDV [1]. This subsequently leads to the development of T lymphomas in peripheral nerves and visceral organs, which induces immunosuppression in MD chickens [1]. Some MDV virulence factors, such as Meq, vTR, vIL-8, ICP4, PP38, UL36, UL49, and microRNAs [4,5,6,7,8], are known to play important roles in the MDV pathogenicity, deletion, or mutation of some MDV-encoded genes (e.g., UL36, UL48, and US2), impacting the MDV replication and horizontal transmission [9], but a single deletion of most genes could not repress the pathogenesis and spread of MDV [10]. Thus, the mechanisms underlying the MDV oncogenicity and immunosuppression, as well as the function of other MDV-associated factors, have not been fully elucidated.

The method of omics analysis has revealed systemic alterations during virus pathogenesis, such as changes to the epigenome, lipidome, proteome, transcriptome, ubiquitylome, and metabolome [11,12]. The omics research on MDV has revealed profile changes in cellular transcriptomes and proteomes in MD-infected chickens [13,14,15]. However, no ubiquitylome study has been reported on MD in chickens.

As one of the most important post-translational modification mechanisms, ubiquitination and deubiquitination regulate almost all cellular processes, including the cell cycle, proliferation, differentiation, apoptosis, chromatin remodeling, transcription, and viral infection, through catalysis by the ubiquitin (Ub) activation enzyme E1, conjugating enzyme E2 and ligase E3, and deubiquitinase (DUB) [16,17,18,19]. Some viruses encode ubiquitin-like modifiers, viral-type ubiquitin E3 ligase, and DUBs. For example, HSV-1 encoded E3 ligase ICP0 and DUB UL36, UL36 of MDV, UL48 of HCMV, Orf64 of KSHV, and BPLF1 of EBV [20]. Viruses utilize these products to hijack the cellular ubiquitination regulatory machinery to promote viral infection, replication, pathogenicity, and genome integration [20]. MDV-encoded UL36, an approximately 360-kDa DUB, is reportedly important for MDV replication, pathogenesis, and shedding [7,21]. Our previous study also confirmed that UL36, which was encoded by the MDV-1 strain J-1, had deubiquitinase activities [22]. It was observed that UL36 was expressed at high levels in T lymphoma cells induced by the strain J-1 MDV in the preliminary work of this study. Thus, it would be interesting to examine alterations in the ubiquitylome of MDV-induced T lymphoma cells. In the current study, a monoclonal antibody against a ubiquitinated peptide (K-ε-GG) was used to isolate ubiquitinated peptides of T lymphoma cells and CD4^+^ T cells. Quantitative and qualitative analyses were performed by the mass spectrometry and bioinformatics analysis. The ubiquitination-related alterations of some proteins were verified by immunoblotting.

## 2. Results and Discussion

### 2.1. Quantification of Ubiquitinated Peptides and Annotation of Proteins

Compared to purified CD4+ T cells, it was found that MDV-encoded DUB, UL36, is highly expressed in MD T lymphoma cells (Figures S1 and S2). According to this observation, the high quality peptides were isolated and analyzed to determine the ubiquitinated Lys sites and the amino acid composition of ubiquitinated peptides (Figures S3 and S4). A total of 7097 ubiquitinated peptides from 2350 proteins was accurately quantitated. Of those, a subtotal of 1638 ubiquitinated peptides in 717 proteins were upregulated (T lymphoma cell/control T cell (T/C) ratios were above 2.0), and 1553 ubiquitinated peptides in 778 proteins were downregulated (control T cells/T lymphoma cells (C/T) ratios were above 2.0). The complete list of proteins with significantly up- and downregulated ubiquitination detected in the T lymphoma cells is given in the Table S1.

### 2.2. MDV Up- and Downregulated Ubiquitination of Two Different Sets of Proteins with Similar Ratios

**Figure 1:**
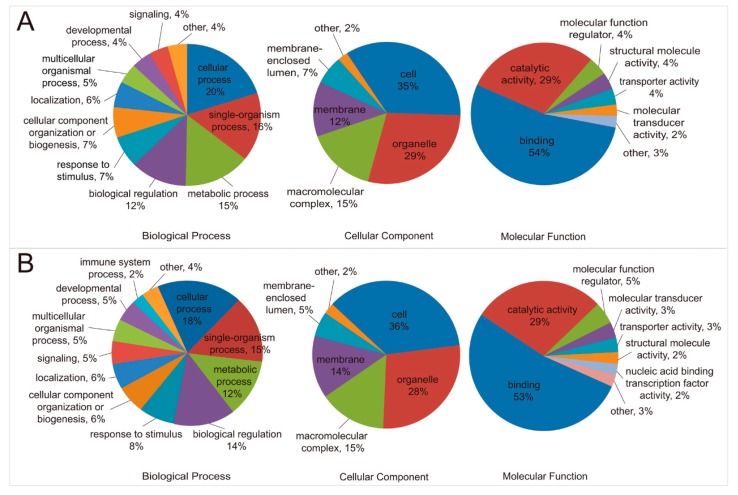
Annotation of significantly changed proteins. Significantly upregulated (**A**) and downregulated (**B**) proteins were classified into three categories each: Biological process, cellular component, and molecular function. The ratio pie charts show the percentages of proteins for each of the categories within the up- and downregulated ubiquitination groups.

As illustrated in Figure 1 and Table S2, some proteins classified in the biological process, cellular component, and molecular function groups were upregulated in ubiquitination by MDV, while similar proportions of other proteins in each group were downregulated in ubiquitination. These results suggested that MDV induces tumorigenesis by reversing the normal CD4^+^ T-cell ubiquitylome by altering the ubiquitination of distinct proteins in the same cellular processes. This observation may imply that preventing the reversal of ubiquitylome of T cells is one of the ways to inhibit MDV pathogenesis.

**Figure 2:**
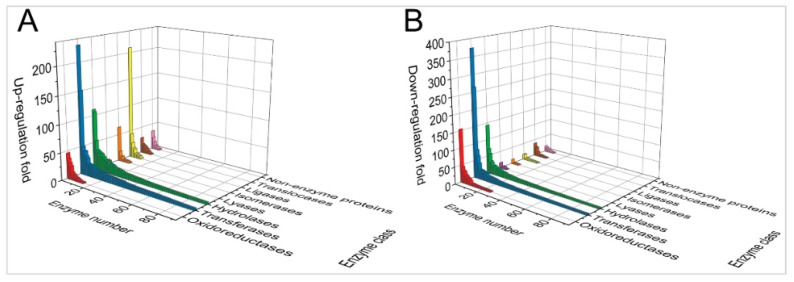
Classification of the proteins related to the catalytic activity. 3D bar graph shows the fold change, number, and classes of upregulated (**A**) or downregulated (**B**) proteins associated with catalytic activities. Each colored bar refers to one protein, and the height of each bar refers to the maximum up- or downregulation fold value of each protein.

More than 80% of the proteins of the molecular function group were associated with catalytic activity and molecular binding (Figure 1). More than 50% of the proteins with a significant ubiquitination alteration were involved with molecular binding. This suggests that MDV might have impacted molecular interactions by alterations in ubiquitination (Figure 1 “Molecular Function”). The proteins associated with the catalytic activity were classified further into different enzyme classes (Figure 2). The ubiquitination of transferases and hydrolases exhibited significantly higher up- and downregulation in contrast to the other enzymes. This suggests that these two classes of enzymes were heavily impacted by MDV, and the function of the upregulated and downregulated enzymes in the process of the MDV tumorigenesis might be opposite functionally. In the same enzyme class, the MDV upregulated enzymes were different from those downregulated by MDV (Table S3). The proteins with a change greater than 100 folds mainly belong to the oxidoreductase, transferase, hydrolase, and ligase families (Figure 2 and Table S3), including upregulated fatty acid synthase (UniProt ID#: P12276), asparagine synthetase (UniProt ID#: Q5ZJU3), CDK1 (cyclin-dependent kinase 1, UniProt ID#: P13863), MCM4 (UniProt ID#: E1C2U4), and PSMC5 (UniProt ID#: F1NU79) and downregulated ZAP70 (zeta-chain-associated protein kinase 70, UniProt ID#: E1BU42), BTK (Bruton’s tyrosine kinase, UniProt ID#: Q8JH64), GPX1 (Glutathione peroxidase 1, UniProt ID#: R4GH86), and ESD (S-formylglutathione hydrolase, UniProt ID#: E1BXC2) (Table S3). As indicated by Figure 1 and Figure 2, MDV may modulate interactions and enzymatic activities of proteins, cellular metabolism, signaling transduction, and so forth, through ubiquitination alteration. The altered cellular homeostasis would eventually promote the transformation of CD4^+^ T cells.

### 2.3. “RD.K..N” Motif Is a Major Regulatory Target of MDV

**Figure 3:**
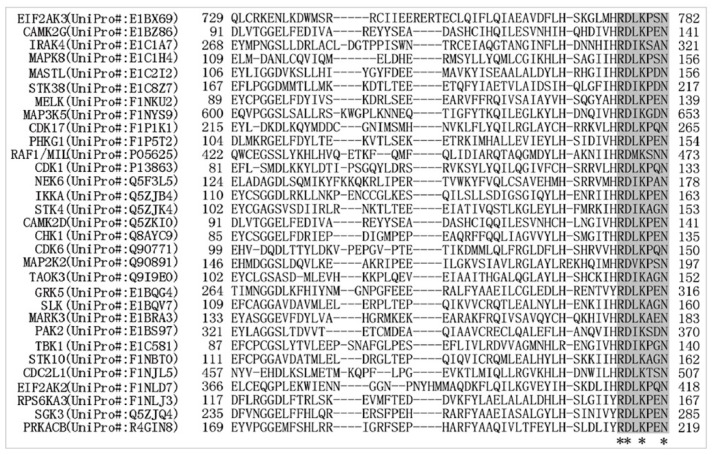
Alignment of proteins containing the “RD.K..N” motif. Alignment of the RD.K..N motifs in various proteins. The RD.K..N motif is highlighted in grey, and asterisks indicate highly conserved residues. Abbreviated protein names are on the left of the parentheses, with the corresponding UniProt ID number shown.

The ubiquitination of 10 types of motifs was found to be significantly up- or downregulated in the proteins examined. The RD.K..N motif exhibited the highest score (Table 1). Among these proteins, the ribosomal protein S6 kinase A3 (UniProt ID#: F1NLJ3) contained two significantly ubiquitinated RD.K..N motifs, located at K167 and K513 (Table S4). Two of those proteins, serine/threonine-protein kinase TAO3 (UniProt ID#: Q9I9E0) and p21 (RAC1)-activated kinase 2 (UniProt ID#: E1BS97), only harbored the ubiquitinated RD.K..N motifs. In addition to the RD.K..N motif, there were also other motifs that were distributed in one or more of the proteins (Table S5). The enzyme classification showed that all proteins that harbored the RD.K..N motif belongs to the serine/threonine kinase family (Table S4). The sequence alignment showed that the RD.K..N motif was highly conserved in all identified kinases (Figure 3). Serine/threonine kinases are among the most important regulators; they function in the T-cell proliferation, apoptosis, and cell differentiation and affect the immune system [23]. The high proportion of ubiquitination alteration that occurred in RD.K..N motifs of T lymphoma cells suggests that these kinases and their participating pathways can be the most important regulatory targets of the MDV infection, and that the RD.K..N motif may play key regulatory roles during the processes. Although the function of ubiquitinated Lys residues in the RD.K..N motif of T-cell serine/threonine kinases remains unclear, further investigation into these enzymes and residues should prove that such studies are worthy of every effort in advancing our understanding of the mechanisms underlying the MDV-induced oncogenesis and immunosuppression.

### 2.4. MDV Up- and Downregulates Two Different Sets of Domains

**Figure 4:**
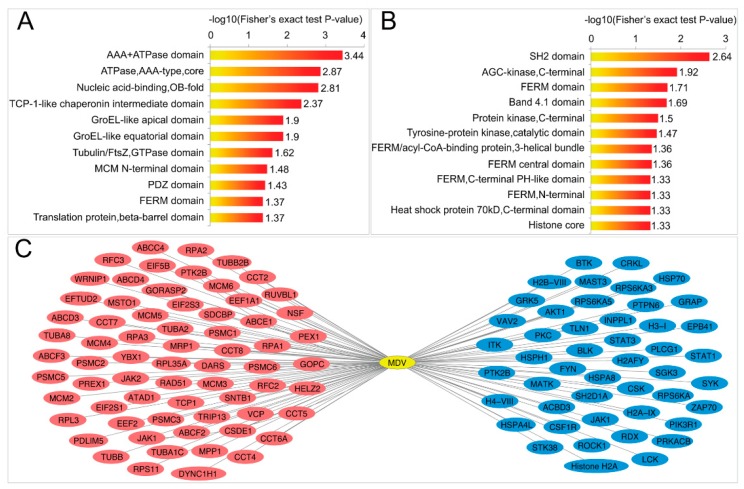
Clustering analysis of domains with *p*-value < 0.05 (Fisher’s exact test). Fisher’s exact test of domains in ubiquitination upregulated (**A**) and downregulated (**B**) proteins. The numbers on the right of the bars are the results of -log10 (Fisher’s exact test *p*-value); (**C**) MDV-induced upregulation (red) and downregulation (blue) in the ubiquitination of protein domains.

The identified domains with *p*-value< 0.05 (Fisher’s exact test) were further analyzed (Figure 4, Figure S5 and Table S6). The ubiquitination of 11 and 12 domains were up- and downregulated, respectively (Figure 4A,B and Table S6). Proteins containing upregulated domains were distinct from those containing downregulated domains (Figure 4C). Both protein groups are involved in important cellular processes, including apoptosis, cell proliferation, and immunoregulation [24,25,26,27,28]. It is likely that the MDV upregulated and downregulated ubiquitination of domains led to alterations of the protein functions, which would ultimately induce the neoplasia and immunologic dysfunction in CD4^+^ T cells.

### 2.5. MDV Mainly Alters Ubiquitination of Proteins Involved in Immune and Cancer Pathways

**Figure 5:**
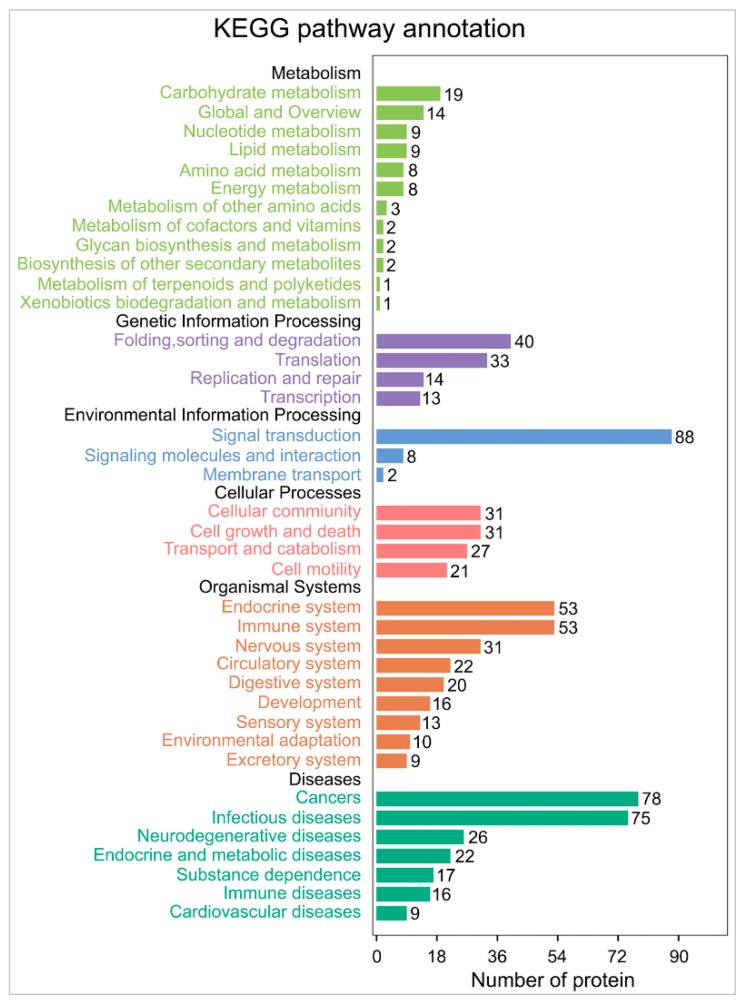
KEGG pathway annotation of proteins with a fold change greater than 10. Annotations in the black font indicate the KEGG pathway A class proteins; other colors indicate KEGG pathway B class proteins. The numbers on the right end of the bars indicate the number of proteins involved in a given pathway.

**Figure 6:**
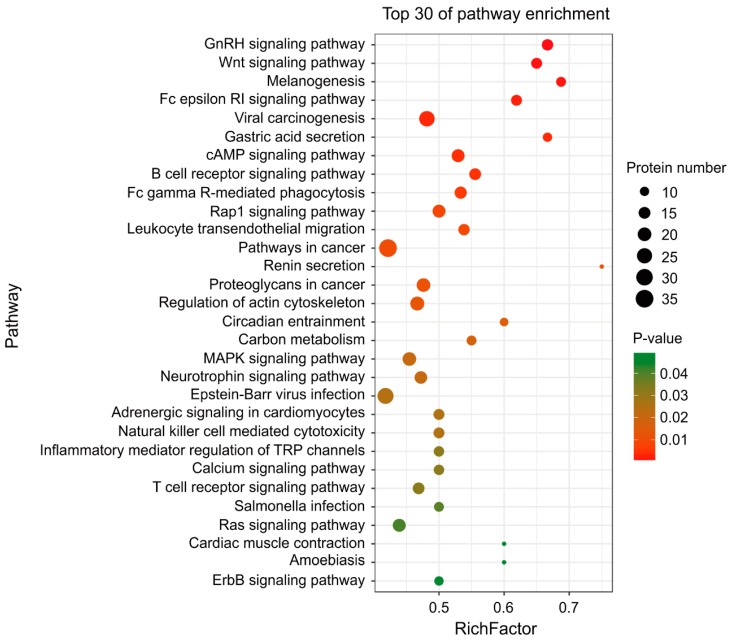
KEGG pathway enrichment analysis using a senior bubble chart. *Y*-axis: Pathway names; *x*-axis: RichFactor (number of proteins belonging to a given pathway/number of all proteins in this given pathway). A greater RichFactor indicates a greater frequency of proteins in a given pathway. The bubble size indicates the number of proteins belonging to a given pathway. The bubble color represents the *p*-value. A color closer to red indicates that there is more convincing evidence of the participation of a protein in a given pathway.

**Figure 7:**
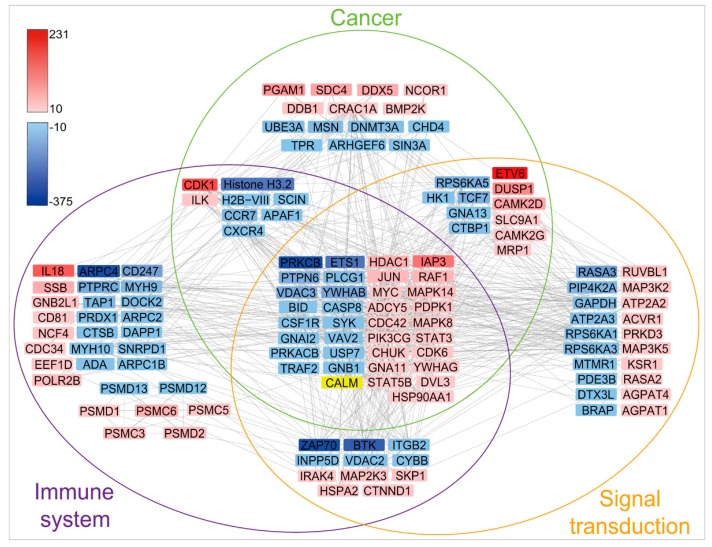
Interaction network of proteins involved in cancer, immune system, and signal transduction pathways. Red indicates upregulated ubiquitination; blue indicates downregulated ubiquitination; yellow indicates simultaneous up- and downregulated ubiquitination at different Lys sites in the CALM protein. Proteins involved in the cancer pathway are encircled by green, proteins associated with the immune system are encircled by purple, and signal transduction proteins are encircled by orange. Proteins involved in two or three pathways are indicated by multiple colored circles. The gray line between two proteins represents interaction. The numbers on the right of the gradient color bars indicates alteration folds, and minus means downregulation; the darker the color (red or blue) of the rectangle, the larger the fold of the ubiquitination alteration (up- or downregulation).

The KEGG pathway annotation indicated that the proteins detected with greater than 10-fold up- or downregulation in ubiquitination participate in a total of 225 pathways (Table S7). The pathways that contain more than 50 proteins with significant ubiquitination alternations are primarily involved in the signal transduction, the endocrine system, the immune system, cancer development, and infectious diseases (Figure 5). A total of 30 top pathways with significant ubiquitination alterations in participated proteins were identified, which are mainly involved in the immune and cancer regulation, including the Wnt signaling pathway, melanogenesis, the Fc epsilon RI signaling pathway, viral carcinogenesis, and the cAMP signaling pathway (Figure 6). Further analyses using String and Cytoscape identified 129 proteins involved in various interaction networks, including signal transduction, the immune system (including infectious disease), and cancer (Figure 7). About 65% of these proteins are involved in immune regulatory pathways, 61% take part in signal transduction pathways, 54% are involved in cancer regulation pathways, and 28% participate in all three pathways. These proteins reportedly play important roles in cell proliferation [29,30,31] and immunoregulation [30,32,33,34] and function as protein kinases (CDK1, PRKCB, ZAP70, BTK, MAP2K3, MAP3K5, BMP2K, etc.), immunoregulators (IL-18, CD81, CD247, etc.), transcription factors (ETV6, ETS1, STAT3, etc.), and regulators of apoptosis (IAP3, JUN, MYC, TRAF2, etc.) (Figure 7).

ZAP70, a protein tyrosine kinase, presents near the surface membrane of normal T cells and natural killer cells. As a subunit of T-cell receptor, ZAP70 plays a crucial role in T-cell activation, differentiation, and signal transduction [35]. BTK is a tyrosine protein kinase, and its mutation results in a primary immunodeficiency disease: X-linked agammaglobulinemia [36]. BTK is very important in maturation and activation of immunocytes [37]. IAP3 (inhibitor of apoptosis protein 3) can inhibit cell apoptosis via downregulation of caspase-3, -7, and -9 through ubiquitination catalyzed by the IAP3 E3 ligase activity [38]. Thus, IAP3 has gained much attention regarding its function in cancer, neurodegenerative disorders, viral infection, and autoimmunity [39]. In addition, IAP3, TRAF2, CASP8, BID, Apaf-1, and so forth, regulate cell apoptosis through the caspase regulation pathway or modulate the expression of virus prosurvival genes through the NF-κB pathway [40]. Thus, the up- and downregulation of ubiquitination on these proteins may antagonize apoptosis and promote T-cell proliferation and MDV intracellular activity. Further exploration of the impact of changes in their ubiquitination might lead to a better understanding of the mechanism of the MDV oncogenesis and immunosuppression.

The abovementioned proteins that are functionally involved in signal transduction, immune system regulation, and cancer pathways accounted for the largest proportion among identified proteins, which suggests that these proteins and pathways are also likely the preferred targets of the MDV infection.

### 2.6. Ubiquitination of Key Proteins Was Significantly Altered in T Lymphoma Cells

**Figure 8:**
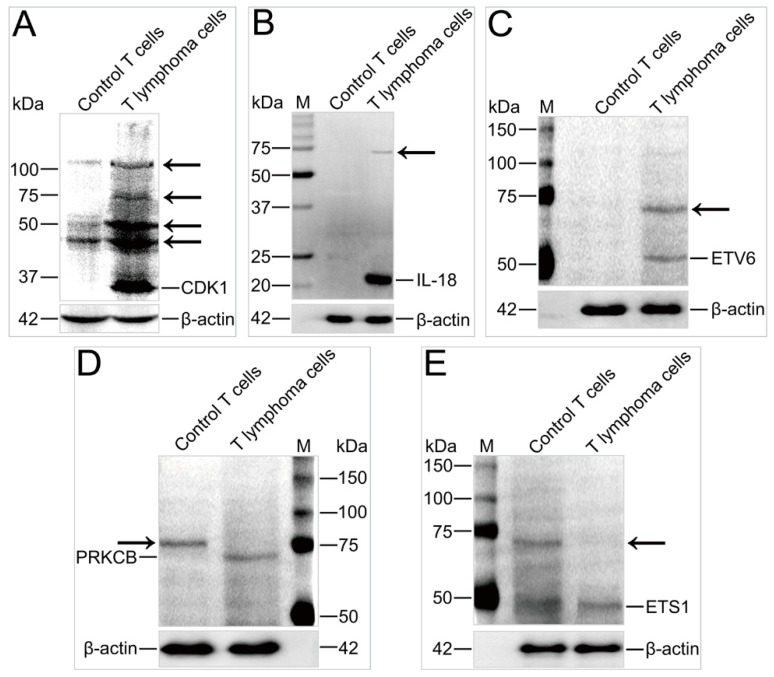
Verification of some identified proteins with immunoblotting. Panels (**A**–**E**) indicate Western blot results detected by primary antibodies against CDK1, IL-18, ETV6, PRKCB, and ETS1, respectively. β-actin bands detected with anti-actin primary antibodies are shown in the lower corner of each panel. Arrows indicate modified protein band(s).

The key targeted proteins of MDV, including kinases (PRKCB and CDK1), cytokines (IL-18), and transcription factors (ETV6 and ETS1), were subjected to immunoblotting to detect changes in ubiquitination. Ubiquitinated PRKCBs were found in control T cells, while unmodified PRKCBs were detected in T lymphoma cells (Figure 8D). It has been reported that PRKCB, a tumor promoter, phosphorylates various cellular proteins and is involved in the immunocyte activation, endothelial cell proliferation, and apoptosis regulation [41]. Our results suggest that the status of the PRKCB ubiquitination would be a contributing factor leading to the T lymphoma cell formation.

Western blots (Figure 8) clearly illustrated the differences of ubiquitinated protein expressions between the control T cells and T lymphoma cells, which included CDK1, IL-18, and ETV6 expressed in higher levels in T lymphoma cells than in the control T cells (Figure 8A–C), and PRKCB and ETS1, vice versa, higher in control T cells (Figure 8D,E). These observations suggest that the MDV infection not only promotes up- and downregulation of ubiquitination but also regulates protein expression levels. CDK1 phosphorylates multiple target proteins to promote cell cycle progression [42]. ETV6 is a proto-oncogene that regulates the development of various types of cells and participates in DNA repair, cellular apoptosis, and cellular differentiation; mutation in ETV6 can also result in lethal hematological cancers [43,44]. As an interferon-gamma-inducing cytokine, IL-18 stimulates the proliferation and IFN-γ release of CD4^+^ T cells in chickens [45]. Chicken IL-18 has two forms: Precursor (23 kDa) and mature (19.5 kDa) [46]. In mammals, the precursor is inactive and only the mature form exhibits activity [47], but both chicken forms are biologically active [46]. In this study, only one band was detected near 20 kDa. Its molecular weight was greater than 20 kDa, so the detected protein was most likely the precursor form, pro-IL-18 (23 kDa). This was probably because only the precursor form exists in MD tumor cells or because very low levels of mature IL-18 were not detected. This result suggests that pro-chIL-18 is a major form in MD T lymphoma cells. It would be interesting to reveal the function of pro-IL-18 in MD tumor cells. As the MDV infection dramatically changed the ubiquitination of the three key proteins, this suggests that the ubiquitination status of these proteins may be essential in the transformation of T lymphocytes.

As an oncogenic transcription factor, ETS1 is highly expressed in immunocytes and participates in the functional regulation of the immune system and differentiation of immune tissue and cells [48]. The abnormal expression and activity of ETS1 cause multiple defects in the immune system [44,49]. As shown in Figure 8E, ETS1 was downregulated in T lymphoma cells in contrast to the control T cells. In addition, only unmodified ETS1 was detected in the T lymphoma cells, while both modified and unmodified ETS1 was observed in control T cells. Our results implied that the lack of ubiquitination of ETS1 might play important roles in the neoplasia and CD4^+^ T-cell dysfunction.

Although multiple ubiquitinations were found in the mass spectrometry of most identified proteins, only one or fewer modified band(s) were detected by the Western blot. The possible cause was that the confluence of ubiquitinated proteins in each cell was low, and Western blot detection sensitivity is much lower than the mass spectrometry. Therefore, some modified proteins might not have been detected by the Western blot. In addition, MS determined that multiple ubiquitinations occurred in these proteins because the anti-K-ε-GG antibody specifically isolated ubiquitinated peptides but not the modified peptides by Ub-like modifiers [50,51]. However, some proteins also could be modified by Ub-like proteins; therefore, it cannot be excluded that the bands detected by the Western blot may have contained the protein which was conjugated by Ub-like modifiers. In future investigations, immunoprecipitation-grade antibodies against these chicken proteins need to be developed to isolate these modified proteins in a sufficient amount. Further, the MS and Western blot should be carried out to determine which modifier conjugates on these identified proteins.

In summary, the ubiquitylome analysis in the current study indicated that the ubiquitination of proteins induced by the MDV infection may be involved in signal transduction, endocrine system, immune system, cancer, and infectious disease pathways, and those proteins were among the main targets of the MDV infection. The key regulatory point should be the RD.K..N motif in serine/threonine protein kinases. We found that MDV upregulated ubiquitination in a few proteins; and at the same time, it also downregulated ubiquitination in a similar percentage of other proteins. MDV altered the homeostasis of intracellular ubiquitylome, which may have led to tumorigenesis in CD4^+^ T cells. The findings of the current study may provide the basic knowledge to facilitate future studies in this area to examine the mechanisms underlying MDV-induced oncogenesis and immunosuppression in T cells associated with ubiquitination regulation. The ubiquitylome analysis in the current study will promote a better understanding of the host–virus interaction and the study of more effective defenses against MD. MDV very closely shares genomic functional and structural characteristics with some mammalian viruses which encode viral DUBs or ubiquitination regulators, such as Cytomegalovirus (CMV), Epstein–Barr virus (EBV), and herpes simplex virus (HSV) [8,52,53]. Further investigations to explore the function of ubiquitination post-MDV infection may advance knowledge not only of MDV-induced pathogenesis but also of other herpesviruses.

## 3. Materials and Methods

### 3.1. Isolation and Characterization of T Lymphocytes

Chicken spleen CD4^+^ T lymphocytes were isolated following the given protocols of the lymphocyte separation kit (TBD Science, Tianjin, China). Briefly, one-day-old specific-pathogen-free (SPF) White Leghorn chickens (Boehringer Ingelheim Vital Biotec. Co. Ltd., Beijing, China) were challenged with the virulent strain J-1 MDV [54]. Chickens not subjected to the MDV challenge were used as controls. At 30–35 days postinfection (dpi), spleens of the control chickens and splenic tumors of MD chickens were sampled and ground on gauze wires for cell dissociation. Mononuclear cells were isolated from the cell mixture using the chicken spleen lymphocyte separation kit (TBD). The isolated mononuclear cells were incubated on nylon wool columns (Polysciences, Inc., Warrington, PA, USA) to remove adherent cells and to isolate T cells [55]. For the CD4^+^ T-cell purification, isolated T cells were further processed using the EasySep CD4^+^ T-cell positive selection kit (STEMCELL Co., Shanghai, China). Cells were probed with a fluorescein isothiocyanate (FITC)-conjugated antibody against CD4 (SouthernBiotech, Birmingham, AL, USA) according to the manufacturer’s instructions. Anti-CD3 antibodies (SouthernBiotech) were added to the eluted CD4^+^ T cells that were bound to FITC-anti-CD4 antibodies. Cells were centrifuged and washed with PBS buffer (10 mM Na_2_HPO_4_, 2 mM KH_2_PO_4_, 137 mM NaCl, 2.7 mM KCl, pH 7.4) to remove unbound antibodies. Purified normal CD4^+^ T cells were used as control T cells, and purified CD4^+^ MD T lymphoma cells were used as the MD tumor cell in the model. A BD Accuri C6 flow cytometer (BD Biosciences, San Jose, CA, USA) was used to assess percentages of CD3 or CD4 antibody-labeled T cells.

### 3.2. Protein Extraction and Immunoaffinity Enrichment of Ubiquitinated Peptides

The protein and ubiquitinated peptide extractions were done as described by Chen et al. [56] and Guo et al. [57] with some modifications. Purified control CD4^+^ T cells or T lymphoma cells were sonicated three times in lysis buffer on ice. The insoluble cell debris was eliminated by centrifugation at 21,000× *g* and 4 °C for 30 min. The proteins in the supernatant were precipitated with chilled 15% trichloroacetic acid (TCA) for 3 h at −20 °C. After centrifugation, the precipitate was collected and washed three times with cold acetone. The precipitate was redissolved in buffer (8 M urea, 100 mM NH_4_HCO_3_, pH 8.0). The concentration of protein solution was determined using a 2D Quant kit (GE Healthcare Biosciences, Pittsburgh, PA, USA) according to the manufacturer’s instructions.

For protein reduction, the above protein solution was added to 10 mM DTT and then incubated at 37 °C for 1 h. Proteins were alkylated by the addition of 20 mM iodoacetamide (IAA; Sigma-Aldrich Corp., St. Louis, MO, USA) for 1 h at room temperature in the dark. For dilution, the protein sample was added to 100 mM NH_4_HCO_3_ and urea at a concentration less than 2 M. Trypsin was added at a 1:50 trypsin-to-protein mass ratio at 37 °C for 12 h for the first-time protein digestion and at a 1:100 trypsin-to-protein mass ratio at 37 °C for 4 h for the second time.

High-pH reversed-phase high-performance liquid chromatography (HPLC) and an Agilent 300 Extend C18 column (5-μm particles, 4.6-mm ID, 250-mm length) were used to fractionate the digested proteins. Peptides were eluted using a fraction buffer (10 mM NH_4_HCO_3_, gradient of 2%–60% acetonitrile (ACN), pH 10), with 80 fractions collected over 80 min. The peptides were then pooled together and dried by vacuum centrifugation.

Antibody-affinity enrichment of ubiquitinated peptides was then conducted as described by Chu et al. [58] and Udeshi et al. [59] with slight modifications. Briefly, the dried peptides were dissolved in an NETN buffer (50 mM Tris-HCl, 100 mM NaCl, 1 mM EDTA, 0.5% NP-40, pH 8.0) and gently mixed with anti-K-ε-GG beads (PTM-1104; PTM Biolabs, Hangzhou, ZJ, China) at 4 °C for 12 h for binding. The beads were washed four times with the NETN buffer and twice with ddH_2_O to eliminate unbound peptides. The bound peptides were then eluted with 0.1% trifluoroacetic acid (Sigma-Aldrich), vacuum-dried, and cleaned with C18 ZipTips (EMD Millipore, Billerica, MA, USA).

### 3.3. LC-MS/MS Analysis and Database Search

The LC-MS/MS analysis and database search were carried out as reported by Chen et al. [56] and Guo et al. [57] with some modifications. Briefly, each sample was analyzed in triplicate. The peptides dissolved in 0.1% formic acid (FA; Sigma-Aldrich) were loaded onto a reverse-phase precolumn (Acclaim PepMap 100; Thermo Fisher Scientific, Waltham, MA, USA) and separated with a reverse-phase analytical column (Acclaim PepMap RSLC; Thermo Fisher Scientific). The solvent B gradient (0.1% FA in 98% ACN) was increased from 6% to 22% for 24 min, from 22% to 35% for eight min, then to 80% in five min, and subsequently held at 80% for an additional three min. The flow rate was a constant 300 nL/min. All collected peptides were identified using a Q Exactive Plus hybrid quadrupole-Orbitrap mass spectrometer (Thermo Fisher Scientific). The peptides were analyzed using a nanospray ionization (NSI) source and MS/MS with a Q Exactive Plus (Thermo) coupled online to an ultraperformance liquid chromatograph (UPLC). Peptides were determined using an Orbitrap at a resolution of 70,000 and selected using MS/MS with the normalized collision energy (NCE) set to 30%. Ion fragments were analyzed in the Orbitrap at 17,500 resolution. One MS scan and then 20 MS/MS scans were used alternatively for the top 20 precursor ions, with an ion count exceeding a 5.0 × 10^3^ threshold using dynamic exclusion for 15.0 s in the MS survey scan. The electrospray voltage was 2.0 kV. MS/MS spectra were produced by the accumulation of 5.0 × 10^4^ ions. The *m*/*z* scan range was 350–1800. The quantitative analysis of the LC-MS/MS data was performed by MaxQuant (v1.6.4.0). The tandem mass spectra were searched against the UniProt chicken database linked to the reverse decoy database. The first search range was set to 10 ppm for precursor ions, and the main search range was set to 5 ppm and 0.02 Da for fragment ions. GlyGly groups on Lys were listed as variable modifications. The false discovery rate (FDR) and the minimum modified peptide score were set at <1% and >40, respectively.

### 3.4. Annotation and Classification of Ubiquitinated Proteins

Identified chicken proteins were annotated using the UniProt-GOA database [60]. Alternatively, InterProScan was used to annotate the GO functions of proteins based on the protein sequence alignment method [61]. The names and IDs of proteins that were not characterized in UniProt were determined using the full-length protein sequence blast in the NCBI BLASTP suite. Next, proteins were classified by the GO annotation and WoLF PSORT based on three categories: Biological process, cellular component, and molecular function [62]. Proteins associated with catalytic activities were further classified into seven enzyme families and other nonenzyme proteins using the “enzyme class” field in UniProt [60], MeSH in NCBI, enzyme nomenclature database in Expasy [63], and ExplorEnz [64]. Fold changes were evaluated and used to plot a 3D bar graph in OriginLab 2017 (OriginLab, Northampton, MA, USA). The domain annotation of ubiquitinated peptides was performed with InterProScan, using the InterPro domain database based on the protein sequence alignment method [65].

### 3.5. Profile Analysis of Ubiquitinated Motifs

Motif models of ubiquitinated peptides were analyzed with motif-x on 21-mer sequences harboring 10 amino acids upstream and downstream of the ubiquitinated Lys site [66]. The motif score and fold increase were calculated according to instructions in motif-x [66]. Enrichment of significantly changed proteins against all identified proteins was examined using the two-tailed Fisher’s exact test. A corrected *p*-value < 0.05 was considered to be significant.

### 3.6. Pathway and Social Network Analysis of Ubiquitinated Proteins

The pathway analysis was carried out using OmicShare tools (www.omicshare.com/tools). Protein data with fold changes greater than 10 were used as the foreground; data with fold changes greater than two were used as the background. By combining the *p*-value or *q*-value, the protein number, and the rich factor, a senior bubble chart was plotted using the OmicShare tools to present the distribution of identified proteins in various pathways. Interaction networks of proteins in certain pathways, such as the immune system (including infectious disease), cancer, and signal transduction pathways, were plotted in String [67]. The profiles and associated networks of these proteins were visualized with Cytoscape 3.6 [68].

### 3.7. Verification of Unique Proteins with Immunoblotting

Proteins with significant ubiquitination changes were verified with Western blotting. Standard Western blotting was preformed using primary antibodies against chicken CDK1 (dilution 1:1000, Abcam, Cambridge, MA, USA), chicken ETV6 (1:2000, MyBioSource, Inc., San Diego, CA, USA), chicken IL-18 (1:500, Abcam), PRKCB (1:500, LifeSpan BioSciences, Inc, Seattle, WA, USA), ETS1 (1:300, LifeSpan BioSciences), and β-actin (1:5000, Abcam). HRP-conjugated goat anti-rabbit IgG secondary antibodies (1:5000, OriGene, Rockville, MD, USA) were used for ECL imaging.

## Figures and Tables

**Table 1:** Motif analysis of the identified and quantifiable proteins.

Motif	Motif Score ^2^	Foreground		Background		Fold Increase ^7^
		Matches ^3^	Size ^4^	Matches ^5^	Size ^6^	
..........K.L........ ^1^	16	1255	9647	51,190	494,441	1.26
..........KL.........	15.95	964	8392	39,148	443,251	1.3
........F.K..........	13.75	399	7428	14,597	404,103	1.49
.......RD.K..N.......	45.1	50	7029	234	389,506	11.84
........I.K..........	13.06	492	6979	19,499	389,272	1.41
........L.K..........	11.96	891	6487	40,364	369,773	1.26
........A.K..........	12.82	595	5596	25,931	329,409	1.35
........D.K..........	13.66	456	5001	19,269	303,478	1.44
........V.K..........	12.78	526	4545	23,908	284,209	1.38
..........K...L......	11.6	496	4019	23,564	260,301	1.36

^1^ A dot indicates any amino acid. ^2^ Higher motif scores typically correspond to motifs that are more significant as well as more specific. ^3^ The term “foreground matches” indicates the number of peptides containing a given motif in identified peptides. ^4^ The term “foreground size” indicates all identified peptides. ^5^ The term “background matches” indicates the number of peptides containing a given motif in the database. ^6^ The term “background size” indicates all motif peptides in the database. ^7^ Fold increase = (foreground matches/background matches)/(foreground size/background size).

## Supplementary Materials

Supplementary Materials can be found at https://www.mdpi.com/1422-0067/20/9/2089/s1. References [69,70] are cited in supplementary materials.

## Abbreviations

MD	Marek’s disease
MDV	Marek’s disease virus
Ub	Ubiquitin
DUB	Deubiquitinase
SPF	Specific pathogen free
FITC	Fluorescein isothiocyanate
MS/MS	Tandem mass spectrometry
FA	Formic acid
