# Supplementary material for: Marek’s Disease Virus Regulates the Ubiquitylome of Chicken CD4+ T Cells to Promote Tumorigenesis
Source: Int J Mol Sci. 2019 Apr 28;20(9):2089. doi: 10.3390/ijms20092089 (PMC6539122; doi:10.3390/ijms20092089)
Supplement: Supplementary file 1 [file ijms-20-02089-s001.zip › Supplementary File/Animal Experimental Ethical Inspection.pdf]

附件

吉林大学动物实验伦理福利审查表

The Tab of Animal Experimental Ethical Inspection，JLU

编号(Number of permit): 201602014

申请人填写的相关信息 (Concerned information wrote by applicant)

申请单位 (Name of organization)： College of Animal Science, Jilin University

申请人学历(Education of applicant) Ph.D. 技术职称(Professional title)：Professor 岗位证书编号(Certificate)：220106197202089610

实验名称(Study title)：Marek's Disease Virus Induces Lymphoma by Altering the Ubiquitylome of Chicken T Lymphocytes

实验目的(Aim of experiment)： The isolation of T lymphocytes of chicken spleen

拟进动物情况

动物来源(Source of animal)： Merial, Inc., Beijing

品种品系(Species or strain)：White Leghorn 等级(Grade)：SPF 规格(Specifications)：1-day-old

数量(Number)：30	申请日期(Application date)：15 February, 2016
进驻日期(Entering date)：1st March, 2016	结束日期(Ending date)：1st October, 2016

实验要点,包括实验方法、观测指标、实验结束后处死动物的方法等(Outline of experiments, experimental methods, observational index, executing animal method et. al)：Chicken are killed by inhalation of carbon dioxide. The spleen of chick is removed under aseptic conditions immediately for T cells isolation. After the experiment, the chicken carcasses are treated in a centralized and harmless treatment.

申请人签名(Signature of applicant)：Yongxing Qi 联系电话(Telephone)：+86-13804314800

审查依据 (Inspection contents)

1. 该项目是否必须用实验动物进行实验，即能否用计算机模拟、细胞培养等非生命方法替代动物或用低等动物替代高等动物进行实验(Does laboratory animal must be used in the project? Could other methods such as computer simulation, cell cultivation or using the low-grade animal instead of the high-grade animal)?
2. 表中所填申请人资格和所用动物的品种品系、质量等级、规格是否合适，能否通过改良设计方案或用高质量的动物来减少所用动物的数量(Are the qualification of applicant，species or strain, grade and specifications of animals suitable? Could the quantity of animals be reduced by improving the study design or using high quality animals)?
3. 能否通过改进实验方法、调整实验观测指标、改良处死动物的方法，来优化实验方案、善待动物(Could the study design and animal treatment be refined by ameliorating experimental method, adjusting observational index, executing animal method)?

审查结果(是否同意申请人的实验方案) (Results of inspection)

委员会专家意见 (Study director)：	同意 (Agree)	☑	不同意 (Disagree)	☐	签名 (Signature)	[signature]
吉林大学实验动物福利伦理委员会 (Institutional Animal Care and Use Committee of Jilin University，IACUC)：	同意 (Agree)	☑	不同意 (Disagree)	☐	签章(Stamp)	吉林大学实验动物中心

备注(Supplement)：
