# Supplementary material for: Marek’s Disease Virus Regulates the Ubiquitylome of Chicken CD4+ T Cells to Promote Tumorigenesis
Source: Int J Mol Sci. 2019 Apr 28;20(9):2089. doi: 10.3390/ijms20092089 (PMC6539122; doi:10.3390/ijms20092089)
Supplement: Supplementary file 1 [file ijms-20-02089-s001.zip › Supplementary File/Supplementary data.docx]

**Supplementary data of ‘Marek’s Disease Virus Regulates the Ubiquitylome of Chicken CD4^+^ T Cells to Promote Tumorigenesis’**

**S1. Materials and Methods**

*S1.1 Characterization of UL36 expression in control T cells or T lymphoma cells*

The same number of unlabelled purified CD4^+^ control T cells or MD T lymphoma cells were resuspended in lysis buffer (8 M urea, 2 mM EDTA), 10 mM dithiothreitol (DTT), 1× Protease Inhibitor Cocktail and PR619 and subjected to repeated freeze-thaw cycles for cell lysis. The proteins in the lysates were then detected by western blotting using standard protocols [69] with anti-UL36(323) primary antibody [70] at a dilution of 1:4,000 and a horseradish peroxidase (HRP)-conjugated goat anti-rabbit IgG secondary antibody at a dilution of 1:5,000 (OriGene Technologies, Rockville, MD, USA).

*S1.2 Profile analysis of ubiquitinated motif*

A heat map was plotted to illustrate the distribution of residues around the ubiquitinated Lys site. The distribution of ten motif models for the identified proteins was analysed in Venny 2.1 (http://bioinfogp.cnb.csic.es/tools/venny/index.html).

**S2. Results**

*S2.1 Verification and identification of the purified chicken CD4^+^ T lymphocytes*

The CD3 and CD4 phenotypes of purified CD4^+^ T lymphocytes were characterized using an immunofluorescence-based flow cytometry assay (Figure S1). The results of flow cytometry showed that the purity of CD4^+^ T cells or T lymphoma cells was more than 95% (FITC signal number in quadrant Q2/Input cell number).


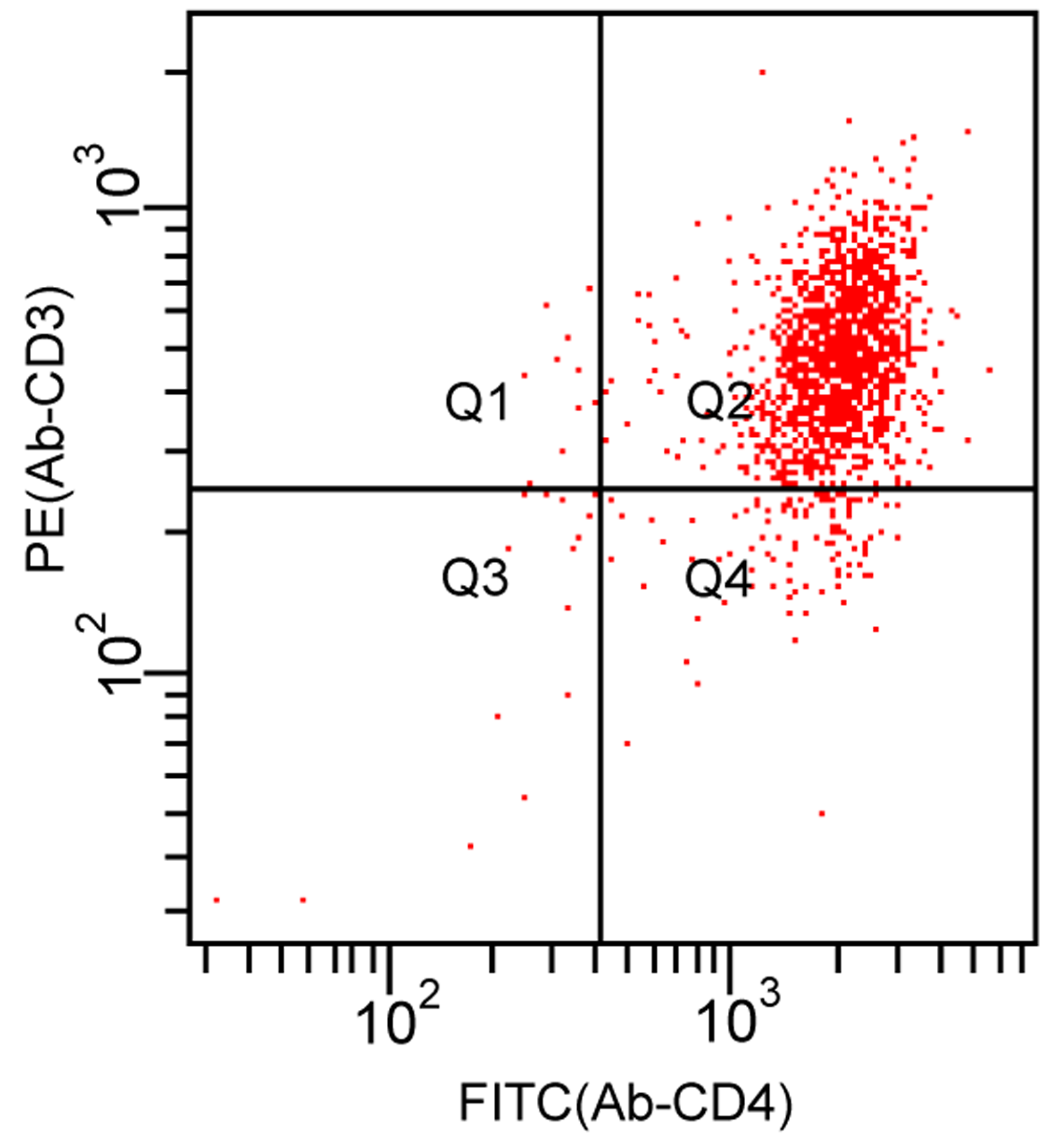


**Figure S1.** Flow cytometry characterization of purified chicken CD4^+^ T cells. The vertical coordinates indicate the fluorescence intensity of the phycoerythrin (PE)-conjugated CD3 antibody, and the horizontal axis indicates the fluorescence intensity of the FITC-conjugated CD4 antibody. The purity of CD4^+^ T cells (FITC signal number in quadrant Q2/Input cell number) was more than 95%.

*S2.2 UL36 is highly expressed in MD T lymphoma cells*

UL36 is a large tegument protein of approximately 360 kDa. The antibody against the N-terminal 323 amino acids of UL36 was employed to UL36 detection in MD T lymphoma cells (Figure S2). Compared with control T cells, UL36 was highly expressed in T lymphoma cells (Figure S2A). The major band was observed at approximately 75 kDa, harbouring the predicted DUB catalytic domain, N-terminus of 323 amino acids. Two minor bands were detected above 180 kDa, suggesting that the full-length UL36 protein exists in T lymphoma cells, though it is not dominant. (Figure S2B).


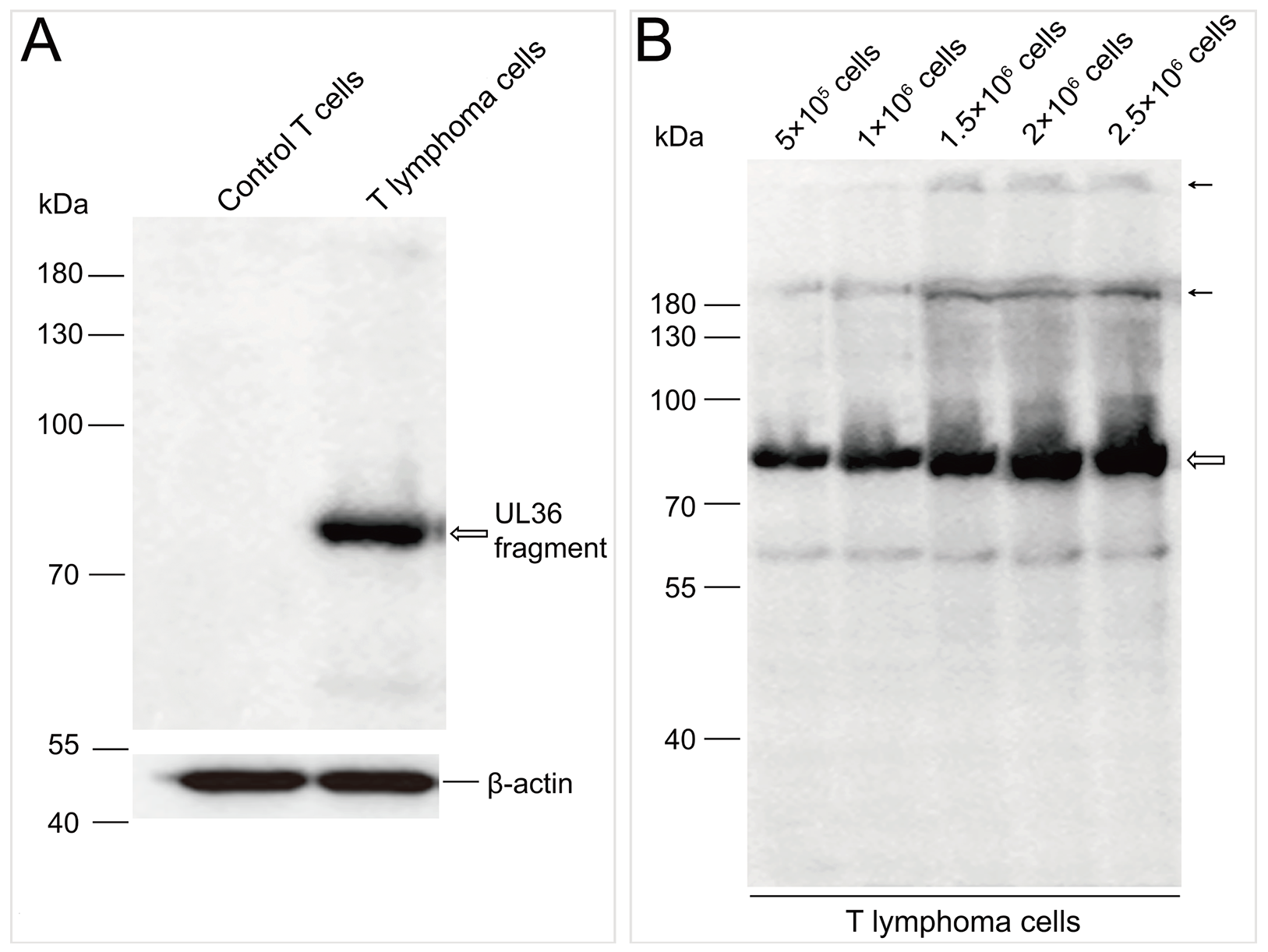


**Figure S2.** UL36 is highly expressed in CD4^+^ T lymphoma cells. (**A**) Comparison of UL36 expression between control T cells and T lymphoma cells. (**B**) Western blot identification of expression levels in T lymphoma cells. The bands indicated by arrows were detected by western blotting with antibody against the UL36-323 protein.

*S2.3 Quantification of ubiquitinated peptides*

The ubiquitinated peptides isolated from T lymphoma cells or control T cells were analysed and quantified via MS. The mass accuracy of the MS data met the requirement because the distribution of the mass error was close to zero and most of the peptides were present at less than 5 ppm (Figure S3A). The lengths of most of the peptides were between 8 and 20 residues, which agrees with the property of tryptic peptides and approaches the standard (Figure S3B).


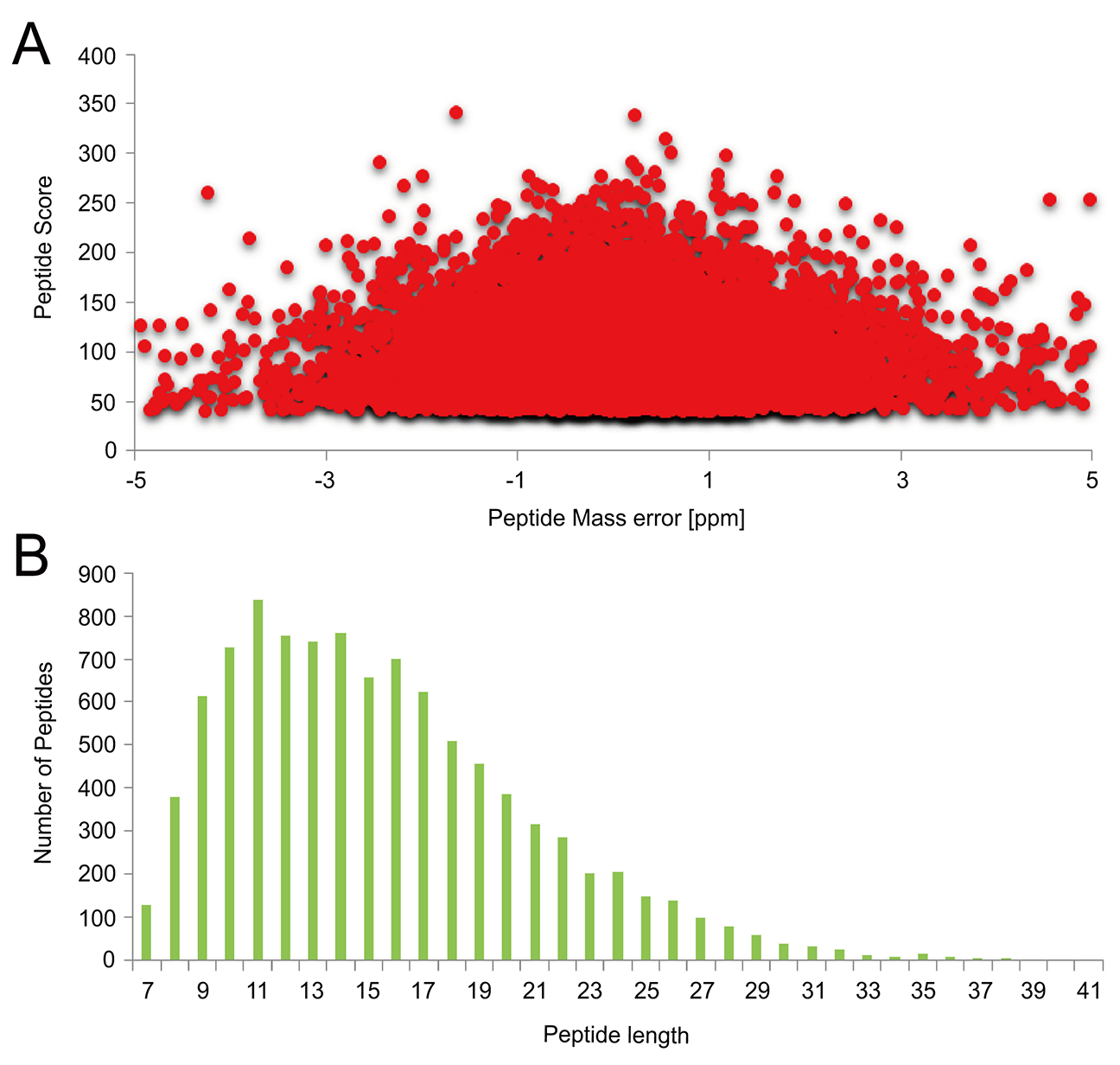


**Figure S3.** Quality control and validation of MS data. (**A**) The distribution of mass error is near zero, and most values are less than 5 ppm, indicating that the mass accuracy of the MS data fit the requirement. (**B**) The length of most peptides is distributed between 8 and 20, which agrees with the property of tryptic peptides.

*S2.4 Flanking residues determine the possibility of ubiquitination on Lys residues*

As presented in Figure S4, in the flanking residues of the ubiquitinated Lys site in modified motifs, nonpolar residues (Ala, Gly, Ile, Leu, and Val), negatively charged residue Asp, and polar uncharged residue Tyr were detected more frequently, Phe and Val were present at sites other than the ±1 sites. Cys was typically found at the +1 site and Asn and Pro at the +3 site of modified Lys. Positively charged residues (His and Lys) and nonpolar residues (Met and Trp) were absent in the identified motifs, and Arg was typically far from the modified Lys site. Other residues were rarely present in the identified motifs. This observation showed that the amino acid composition near a Lys site determines whether ubiquitination and deubiquitination occurs on this Lys residue.


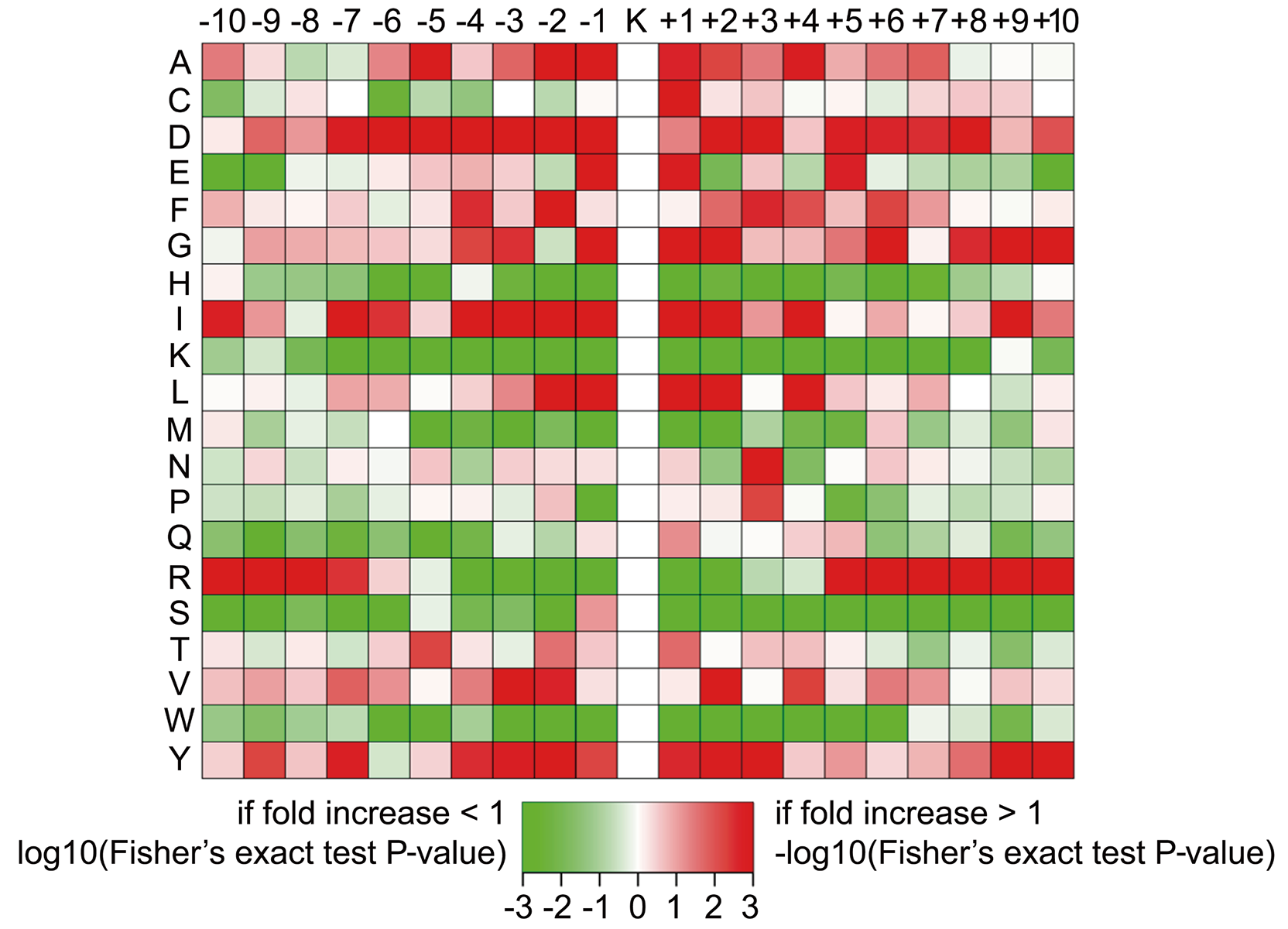


**Figure S4.** Distribution heat map of residues flanked by ubiquitinated Lys sites. The labels on the left of the heat map are the amino acid abbreviations. The top of label ‘K’ indicates the ubiquitinated Lys site, and the flanking numbers indicate the up- or down-stream residue sites.

**The legends of other supplementary figure and tables**

**Figure S5.** Quantile-based clustering for protein domains. The quantifiable ubiquitinated proteins in this study were divided into four quantiles according to T/C ratio: Q1 (0<ratio of T/C <1/4), Q2 (1/4≤ratio of T/C <1/2), Q3 (2<ratio of T/C ≤4) and Q4 (ratio of T/C >4). Next, the quantifiable proteins from the four categories were plotted for protein domain clustering analysis. Z-score = -log10 (Fisher’s test P-value). A deeper red colour indicates that the domain is more likely to be present in the current analysis.

**Table S1.** The list of peptides with significantly up- or down-regulated ubiquitination. Two tables are the list of peptides with significantly up-regulated ubiquitination (**A**) and down-regulated ubiquitination (**B**). T/C Ratio indicates the ratio of T lymphoma cells/control T cells in ubiquitination alteration fold, while C/T Ratio is control T cells / T lymphoma cells. Each P-value was generated from T test of the ratios of three repeated measures. Modified residue was Lys (K). Protein accession is Uniprot accession number.

**Table S2.** Annotation of identified proteins with significantly up- and down-regulated ubiquitination.

**Table S3.** The classification of the significantly changed proteins associated with catalytic activity.

**Table S4.** Annotation of proteins containing the ‘RD.K..N’ motif.

**Table S5.** Distribution analysis of motifs in identified proteins. The number in parentheses behind the motif name represents the score.

**Table S6.** Annotation of domains with significantly up- and down-regulated ubiquitination. Two tables are the list of domains with significantly up-(**A**) and down-(**B**) regulated ubiquitination and a Fisher’s exact test p-value less than 0.05.

**Table S7.** KEGG pathway analysis of proteins with a fold change greater than 10
