# Supplementary figures and images for: Marek’s Disease Virus Regulates the Ubiquitylome of Chicken CD4+ T Cells to Promote Tumorigenesis
Source: Int J Mol Sci. 2019 Apr 28;20(9):2089. doi: 10.3390/ijms20092089 (PMC6539122; doi:10.3390/ijms20092089)

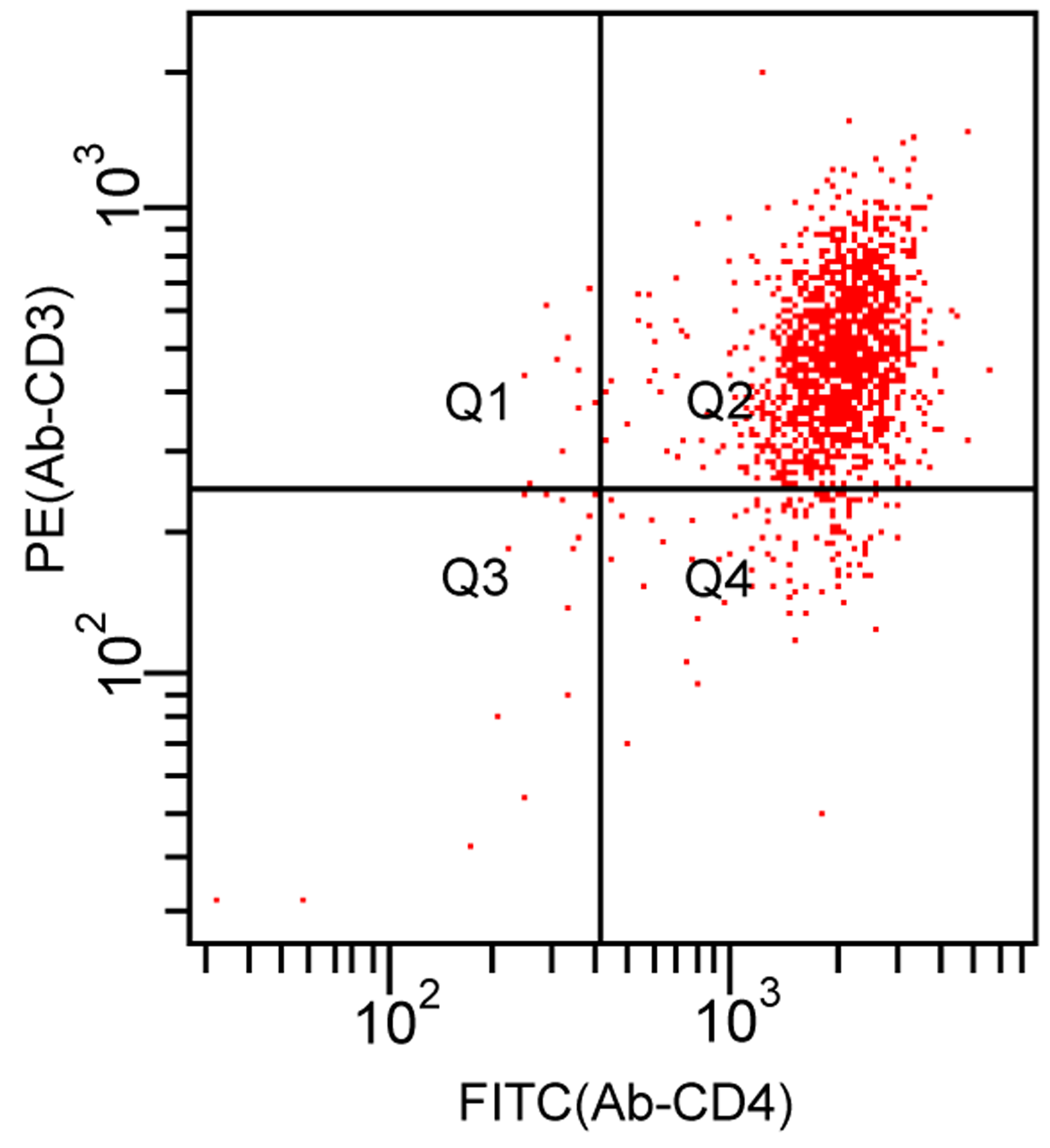

Supplement: Supplementary file 1 [file ijms-20-02089-s001.zip › Supplementary File/Fig S1.tif]

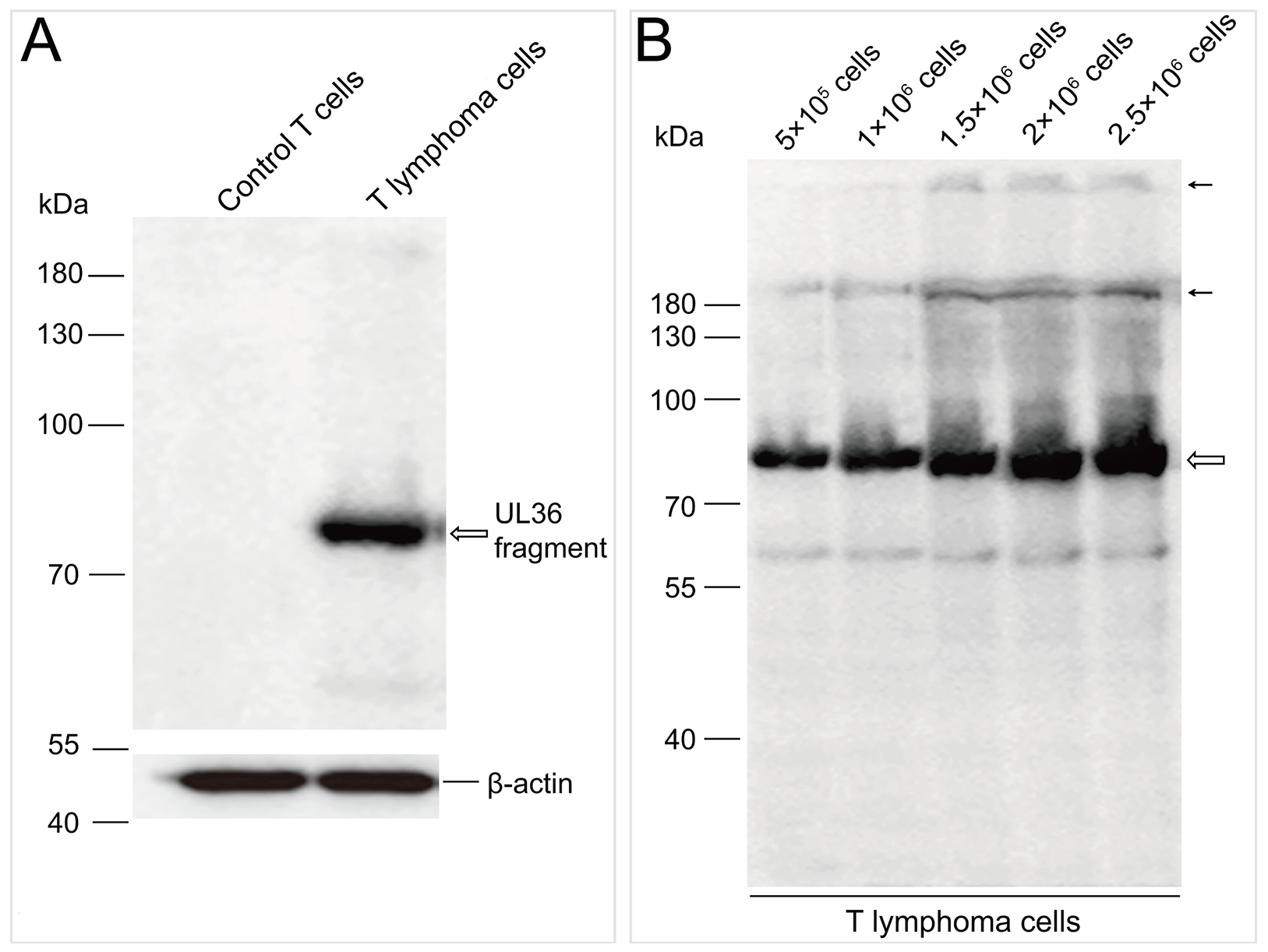

Supplement: Supplementary file 1 [file ijms-20-02089-s001.zip › Supplementary File/Fig S2.tif]

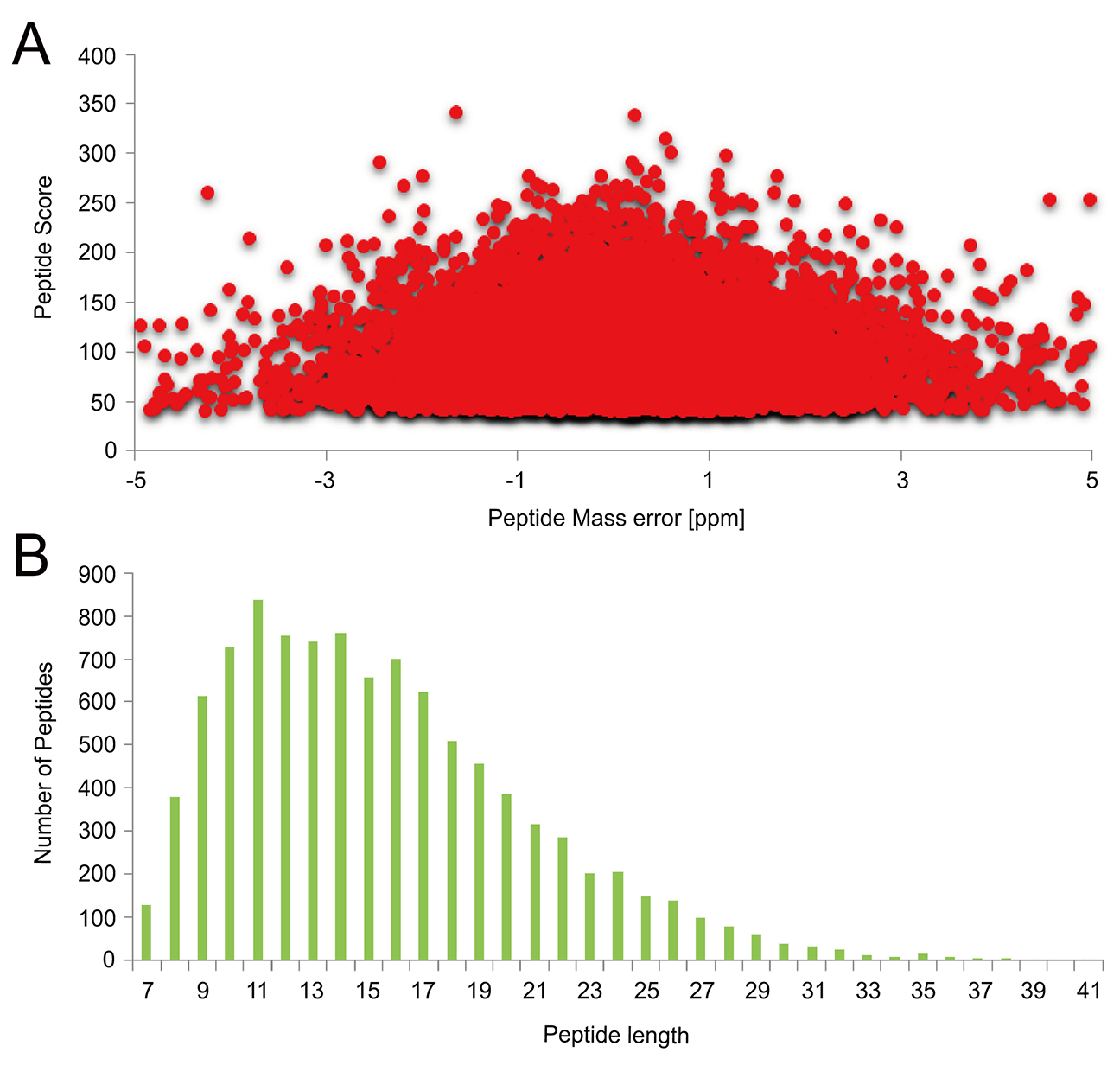

Supplement: Supplementary file 1 [file ijms-20-02089-s001.zip › Supplementary File/Fig S3.tif]

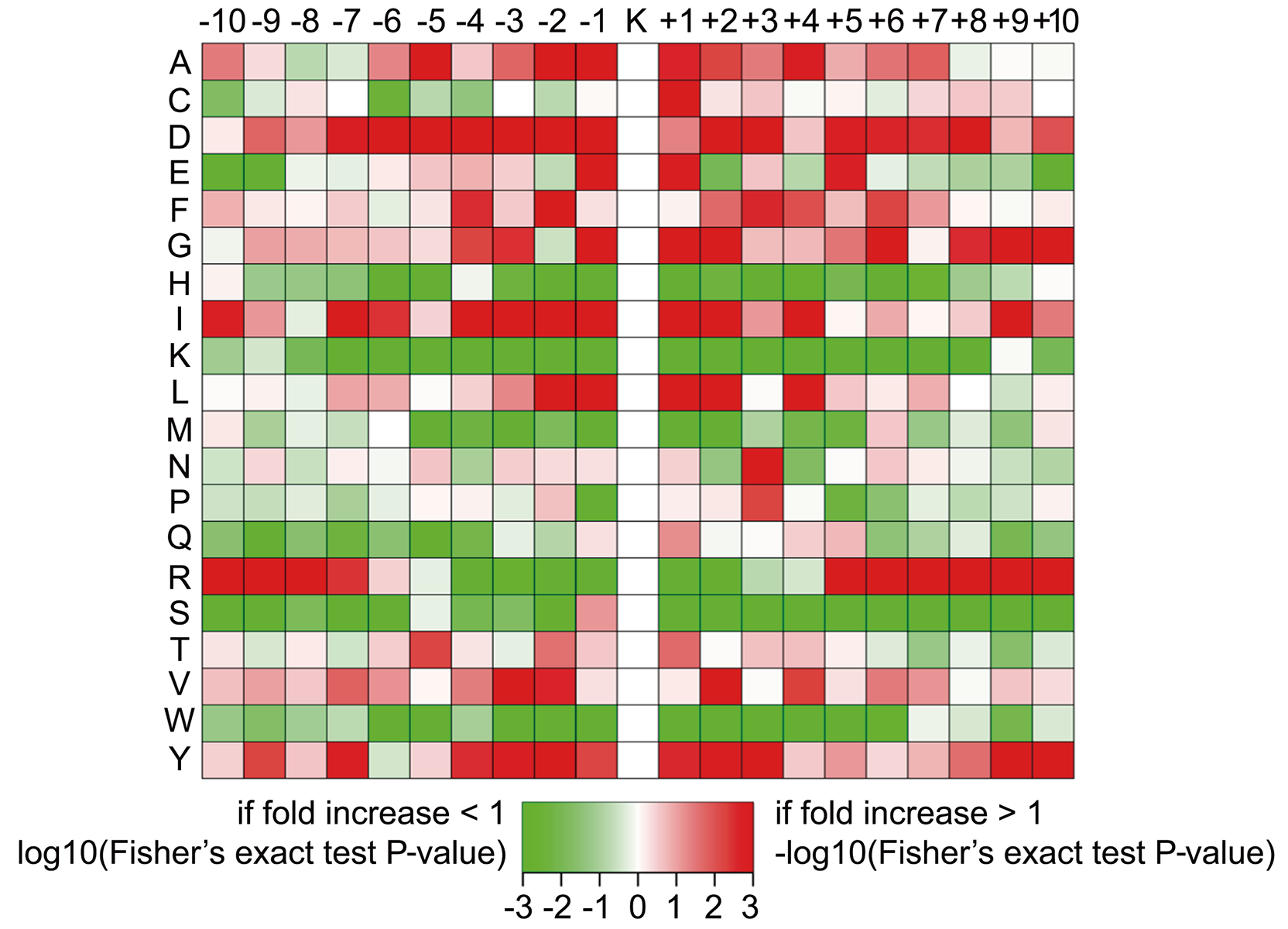

Supplement: Supplementary file 1 [file ijms-20-02089-s001.zip › Supplementary File/Fig S4.tif]

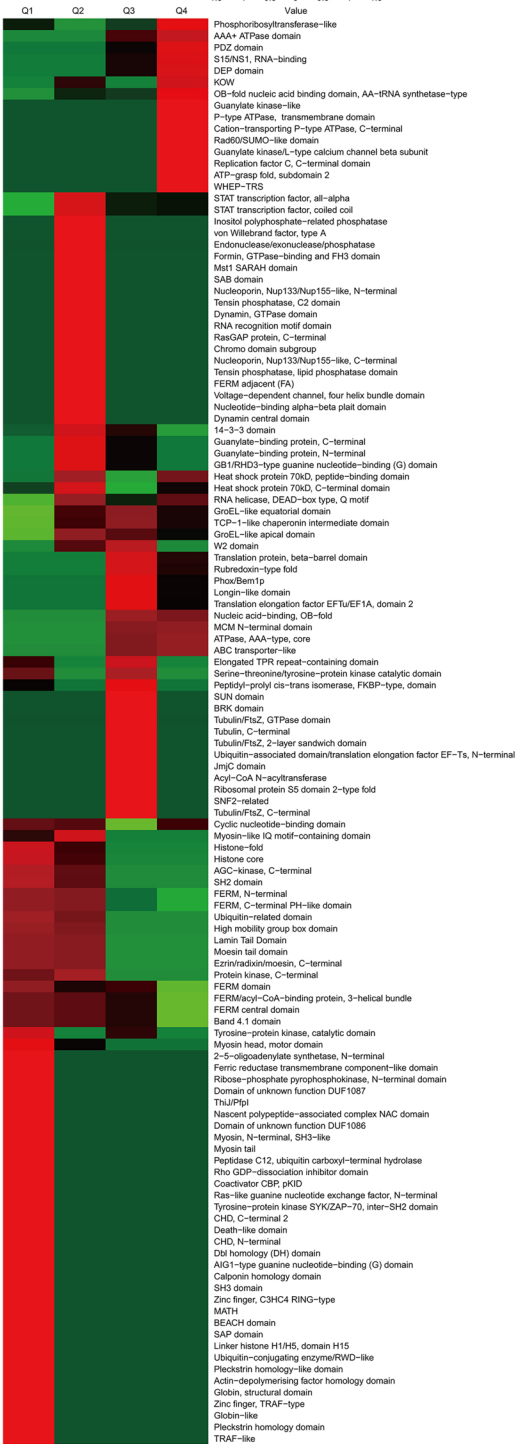

Supplement: Supplementary file 1 [file ijms-20-02089-s001.zip › Supplementary File/Fig S5.pdf]
