# Supplementary material for: Marek’s Disease Virus Regulates the Ubiquitylome of Chicken CD4+ T Cells to Promote Tumorigenesis
Source: Int J Mol Sci. 2019 Apr 28;20(9):2089. doi: 10.3390/ijms20092089 (PMC6539122; doi:10.3390/ijms20092089)
Supplement: Supplementary file 1 [file ijms-20-02089-s001.zip › Supplementary File/Table S5.doc]

Table S5 Distribution analysis of motifs in identified proteins

| **Uniprot ID# of protein** | **Name of the included motif** |
| --- | --- |
| P12276  F1NB20  E1BTX8  E1BR42  P11501  F1NMJ9 | K...L-motif(11.6)  L.K-motif(11.96)  V.K-motif(12.78)  A.K-motif(12.82)  I.K-motif(13.06)  D.K-motif(13.66)  F.K-motif(13.75)  KL-motif(15.95)  K.L-motif(16) |
| P13863 | K...L-motif(11.6)  L.K-motif(11.96)  V.K-motif(12.78)  A.K-motif(12.82)  D.K-motif(13.66)  F.K-motif(13.75)  KL-motif(15.95)  K.L-motif(16)  RD.K..N-motif(45.1) |
| Q5ZMU9  E1BQC5 | K...L-motif(11.6)  L.K-motif(11.96)  V.K-motif(12.78)  A.K-motif(12.82)  I.K-motif(13.06)  D.K-motif(13.66)  F.K-motif(13.75)  K.L-motif(16) |
| Q5ZMG9  Q5ZIT7  E1C2F9 | K...L-motif(11.6)  L.K-motif(11.96)  V.K-motif(12.78)  A.K-motif(12.82)  I.K-motif(13.06)  D.K-motif(13.66)  KL-motif(15.95)  K.L-motif(16) |
| P14105 | K...L-motif(11.6)  L.K-motif(11.96)  V.K-motif(12.78)  A.K-motif(12.82)  D.K-motif(13.66)  F.K-motif(13.75)  KL-motif(15.95)  K.L-motif(16) |
| F1P1K1 | K...L-motif(11.6)  L.K-motif(11.96)  V.K-motif(12.78)  A.K-motif(12.82)  D.K-motif(13.66)  KL-motif(15.95)  K.L-motif(16)  RD.K..N-motif(45.1) |
| F1NEF6 | K...L-motif(11.6)  L.K-motif(11.96)  V.K-motif(12.78)  I.K-motif(13.06)  D.K-motif(13.66)  F.K-motif(13.75)  KL-motif(15.95)  K.L-motif(16) |
| E1BQU4  F1P575 | K...L-motif(11.6)  L.K-motif(11.96)  A.K-motif(12.82)  I.K-motif(13.06)  D.K-motif(13.66)  F.K-motif(13.75)  KL-motif(15.95)  K.L-motif(16) |
| Q5ZJJ2  Q9YGL9 | K...L-motif(11.6)  V.K-motif(12.78)  A.K-motif(12.82)  I.K-motif(13.06)  D.K-motif(13.66)  F.K-motif(13.75)  KL-motif(15.95)  K.L-motif(16) |
| Q5ZMC2  F1NCB0 | L.K-motif(11.96)  V.K-motif(12.78)  A.K-motif(12.82)  I.K-motif(13.06)  D.K-motif(13.66)  F.K-motif(13.75)  KL-motif(15.95)  K.L-motif(16) |
| Q5ZJK4 | L.K-motif(11.96)  V.K-motif(12.78)  A.K-motif(12.82)  I.K-motif(13.06)  F.K-motif(13.75)  KL-motif(15.95)  K.L-motif(16)  RD.K..N-motif(45.1) |
| Q5ZKL0 | K...L-motif(11.6)  L.K-motif(11.96)  V.K-motif(12.78)  A.K-motif(12.82)  I.K-motif(13.06)  D.K-motif(13.66)  KL-motif(15.95) |
| F1NT80 | K...L-motif(11.6)  L.K-motif(11.96)  V.K-motif(12.78)  A.K-motif(12.82)  I.K-motif(13.06)  D.K-motif(13.66)  K.L-motif(16) |
| F1NAG0  E1C8F6  Q8JH64 | K...L-motif(11.6)  L.K-motif(11.96)  V.K-motif(12.78)  A.K-motif(12.82)  I.K-motif(13.06)  F.K-motif(13.75)  K.L-motif(16) |
| F1NK47 | K...L-motif(11.6)  L.K-motif(11.96)  V.K-motif(12.78)  A.K-motif(12.82)  I.K-motif(13.06)  KL-motif(15.95)  K.L-motif(16) |
| F1NB03 | K...L-motif(11.6)  L.K-motif(11.96)  V.K-motif(12.78)  A.K-motif(12.82)  D.K-motif(13.66)  KL-motif(15.95)  K.L-motif(16) |
| P14732 | K...L-motif(11.6)  L.K-motif(11.96)  V.K-motif(12.78)  A.K-motif(12.82)  F.K-motif(13.75)  KL-motif(15.95)  K.L-motif(16) |
| E1C2F7  Q04619 | K...L-motif(11.6)  L.K-motif(11.96)  V.K-motif(12.78)  I.K-motif(13.06)  D.K-motif(13.66)  KL-motif(15.95)  K.L-motif(16) |
| Q5ZIX8 | K...L-motif(11.6)  L.K-motif(11.96)  V.K-motif(12.78)  I.K-motif(13.06)  F.K-motif(13.75)  KL-motif(15.95)  K.L-motif(16) |
| Q5ZJK8 | K...L-motif(11.6)  L.K-motif(11.96)  V.K-motif(12.78)  D.K-motif(13.66)  F.K-motif(13.75)  KL-motif(15.95)  K.L-motif(16) |
| Q5F3L5 | K...L-motif(11.6)  L.K-motif(11.96)  V.K-motif(12.78)  D.K-motif(13.66)  KL-motif(15.95)  K.L-motif(16)  RD.K..N-motif(45.1) |
| E1C8W4 | K...L-motif(11.6)  L.K-motif(11.96)  A.K-motif(12.82)  I.K-motif(13.06)  D.K-motif(13.66)  KL-motif(15.95)  K.L-motif(16) |
| Q5ZM36 | K...L-motif(11.6)  L.K-motif(11.96)  A.K-motif(12.82)  I.K-motif(13.06)  F.K-motif(13.75)  KL-motif(15.95)  K.L-motif(16) |
| E1C2I2 | K...L-motif(11.6)  L.K-motif(11.96)  A.K-motif(12.82)  D.K-motif(13.66)  F.K-motif(13.75)  KL-motif(15.95)  RD.K..N-motif(45.1) |
| P24798 | K...L-motif(11.6)  V.K-motif(12.78)  A.K-motif(12.82)  I.K-motif(13.06)  D.K-motif(13.66)  KL-motif(15.95)  K.L-motif(16) |
| E1C8H5  Q5ZJX6  F1NBL6 | L.K-motif(11.96)  V.K-motif(12.78)  A.K-motif(12.82)  I.K-motif(13.06)  D.K-motif(13.66)  KL-motif(15.95)  K.L-motif(16) |
| F1P409  Q5ZM33 | L.K-motif(11.96)  V.K-motif(12.78)  A.K-motif(12.82)  I.K-motif(13.06)  F.K-motif(13.75)  KL-motif(15.95)  K.L-motif(16) |
| F1NLJ3 | L.K-motif(11.96)  I.K-motif(13.06)  D.K-motif(13.66)  F.K-motif(13.75)  KL-motif(15.95)  K.L-motif(16)  RD.K..N-motif(45.1) |
| F1NU79  P00548 | K...L-motif(11.6)  L.K-motif(11.96)  V.K-motif(12.78)  A.K-motif(12.82)  I.K-motif(13.06)  D.K-motif(13.66) |
| R4GJW5 | K...L-motif(11.6)  L.K-motif(11.96)  V.K-motif(12.78)  A.K-motif(12.82)  I.K-motif(13.06)  K.L-motif(16) |
| F1NW23  E1C2U4 | K...L-motif(11.6)  L.K-motif(11.96)  V.K-motif(12.78)  A.K-motif(12.82)  D.K-motif(13.66)  K.L-motif(16) |
| E1C470  Q5ZLW7  F1NAG9 | K...L-motif(11.6)  L.K-motif(11.96)  V.K-motif(12.78)  A.K-motif(12.82)  KL-motif(15.95)  K.L-motif(16) |
| F1NSP8  E1C8G8  Q5F411 | K...L-motif(11.6)  L.K-motif(11.96)  V.K-motif(12.78)  I.K-motif(13.06)  KL-motif(15.95)  K.L-motif(16) |
| E1C3X1 | K...L-motif(11.6)  L.K-motif(11.96)  V.K-motif(12.78)  F.K-motif(13.75)  KL-motif(15.95)  K.L-motif(16) |
| Q5ZLQ6  Q5ZMD1 | K...L-motif(11.6)  L.K-motif(11.96)  A.K-motif(12.82)  I.K-motif(13.06)  D.K-motif(13.66)  KL-motif(15.95) |
| Q5ZLE6  P09654 | K...L-motif(11.6)  L.K-motif(11.96)  A.K-motif(12.82)  I.K-motif(13.06)  KL-motif(15.95)  K.L-motif(16) |
| Q5ZJU3 | K...L-motif(11.6)  L.K-motif(11.96)  A.K-motif(12.82)  D.K-motif(13.66)  F.K-motif(13.75)  K.L-motif(16) |
| Q5ZKI7  E1BTC3  F6R1X6 | K...L-motif(11.6)  L.K-motif(11.96)  A.K-motif(12.82)  F.K-motif(13.75)  KL-motif(15.95)  K.L-motif(16) |
| Q75R65 | K...L-motif(11.6)  L.K-motif(11.96)  I.K-motif(13.06)  F.K-motif(13.75)  KL-motif(15.95)  K.L-motif(16) |
| Q90771 | K...L-motif(11.6)  L.K-motif(11.96)  I.K-motif(13.06)  KL-motif(15.95)  K.L-motif(16)  RD.K..N-motif(45.1) |
| Q90988 | K...L-motif(11.6)  V.K-motif(12.78)  A.K-motif(12.82)  I.K-motif(13.06)  D.K-motif(13.66)  KL-motif(15.95) |
| E1BX02 | K...L-motif(11.6)  V.K-motif(12.78)  A.K-motif(12.82)  I.K-motif(13.06)  D.K-motif(13.66)  K.L-motif(16) |
| F1ND53  F1NPA9 | K...L-motif(11.6)  V.K-motif(12.78)  A.K-motif(12.82)  I.K-motif(13.06)  KL-motif(15.95)  K.L-motif(16) |
| F1NYS9 | K...L-motif(11.6)  V.K-motif(12.78)  A.K-motif(12.82)  KL-motif(15.95)  K.L-motif(16)  RD.K..N-motif(45.1) |
| Q5ZKX2 | K...L-motif(11.6)  V.K-motif(12.78)  I.K-motif(13.06)  D.K-motif(13.66)  F.K-motif(13.75)  K.L-motif(16) |
| Q5ZLT7 | K...L-motif(11.6)  A.K-motif(12.82)  I.K-motif(13.06)  F.K-motif(13.75)  KL-motif(15.95)  K.L-motif(16) |
| Q90705 | L.K-motif(11.96)  V.K-motif(12.78)  A.K-motif(12.82)  I.K-motif(13.06)  F.K-motif(13.75)  KL-motif(15.95) |
| E1BT08 | L.K-motif(11.96)  V.K-motif(12.78)  A.K-motif(12.82)  I.K-motif(13.06)  F.K-motif(13.75)  K.L-motif(16) |
| P14731  Q805F9  P41239 | L.K-motif(11.96)  V.K-motif(12.78)  A.K-motif(12.82)  I.K-motif(13.06)  KL-motif(15.95)  K.L-motif(16) |
| Q5ZLP8  Q5ZM16  F1N9U8 | L.K-motif(11.96)  V.K-motif(12.78)  A.K-motif(12.82)  D.K-motif(13.66)  KL-motif(15.95)  K.L-motif(16) |
| F1NLD7 | L.K-motif(11.96)  V.K-motif(12.78)  I.K-motif(13.06)  F.K-motif(13.75)  K.L-motif(16)  RD.K..N-motif(45.1) |
| F1NMS0 | L.K-motif(11.96)  V.K-motif(12.78)  D.K-motif(13.66)  F.K-motif(13.75)  KL-motif(15.95)  K.L-motif(16) |
| O93378  Q5F364 | L.K-motif(11.96)  A.K-motif(12.82)  I.K-motif(13.06)  F.K-motif(13.75)  KL-motif(15.95)  K.L-motif(16) |
| F1NWL9 | V.K-motif(12.78)  A.K-motif(12.82)  I.K-motif(13.06)  D.K-motif(13.66)  KL-motif(15.95)  K.L-motif(16) |
| P26446 | A.K-motif(12.82)  I.K-motif(13.06)  D.K-motif(13.66)  F.K-motif(13.75)  KL-motif(15.95)  K.L-motif(16) |
| Q90941 | K...L-motif(11.6)  L.K-motif(11.96)  V.K-motif(12.78)  I.K-motif(13.06)  KL-motif(15.95) |
| E1C396 | K...L-motif(11.6)  L.K-motif(11.96)  A.K-motif(12.82)  I.K-motif(13.06)  KL-motif(15.95) |
| Q9DDT2 | K...L-motif(11.6)  L.K-motif(11.96)  A.K-motif(12.82)  I.K-motif(13.06)  K.L-motif(16) |
| O73885 | K...L-motif(11.6)  L.K-motif(11.96)  A.K-motif(12.82)  D.K-motif(13.66)  K.L-motif(16) |
| Q8UWC5  F1NC02 | K...L-motif(11.6)  L.K-motif(11.96)  A.K-motif(12.82)  KL-motif(15.95)  K.L-motif(16) |
| Q5ZKI0 | K...L-motif(11.6)  L.K-motif(11.96)  A.K-motif(12.82)  K.L-motif(16)  RD.K..N-motif(45.1) |
| Q5F478 | K...L-motif(11.6)  L.K-motif(11.96)  I.K-motif(13.06)  F.K-motif(13.75)  K.L-motif(16) |
| F6SG96 | K...L-motif(11.6)  L.K-motif(11.96)  D.K-motif(13.66)  F.K-motif(13.75)  KL-motif(15.95) |
| E1BS79 | K...L-motif(11.6)  L.K-motif(11.96)  D.K-motif(13.66)  F.K-motif(13.75)  K.L-motif(16) |
| F1P1A5 | K...L-motif(11.6)  L.K-motif(11.96)  D.K-motif(13.66)  KL-motif(15.95)  K.L-motif(16) |
| Q5ZJ70  E1C4M0 | K...L-motif(11.6)  L.K-motif(11.96)  F.K-motif(13.75)  KL-motif(15.95)  K.L-motif(16) |
| Q90891 | K...L-motif(11.6)  L.K-motif(11.96)  KL-motif(15.95)  K.L-motif(16)  RD.K..N-motif(45.1) |
| Q5ZLG0 | K...L-motif(11.6)  V.K-motif(12.78)  A.K-motif(12.82)  I.K-motif(13.06)  K.L-motif(16) |
| F1NCR4 | K...L-motif(11.6)  V.K-motif(12.78)  A.K-motif(12.82)  D.K-motif(13.66)  K.L-motif(16) |
| P53033 | K...L-motif(11.6)  V.K-motif(12.78)  A.K-motif(12.82)  F.K-motif(13.75)  KL-motif(15.95) |
| Q5ZMR3  Q5ZI60  Q8UVF8 | K...L-motif(11.6)  V.K-motif(12.78)  A.K-motif(12.82)  KL-motif(15.95)  K.L-motif(16) |
| P0CB50 | K...L-motif(11.6)  V.K-motif(12.78)  I.K-motif(13.06)  D.K-motif(13.66)  F.K-motif(13.75) |
| F1NPP8 | K...L-motif(11.6)  V.K-motif(12.78)  I.K-motif(13.06)  D.K-motif(13.66)  KL-motif(15.95) |
| Q5ZJ00  Q8JFP1 | K...L-motif(11.6)  V.K-motif(12.78)  D.K-motif(13.66)  F.K-motif(13.75)  KL-motif(15.95) |
| F1NIB4 | K...L-motif(11.6)  V.K-motif(12.78)  D.K-motif(13.66)  F.K-motif(13.75)  K.L-motif(16) |
| F1NYJ6  F1NDY2 | K...L-motif(11.6)  A.K-motif(12.82)  I.K-motif(13.06)  KL-motif(15.95)  K.L-motif(16) |
| E1C4M6  P50147  E1C6T4 | K...L-motif(11.6)  A.K-motif(12.82)  D.K-motif(13.66)  KL-motif(15.95)  K.L-motif(16) |
| P13648 | K...L-motif(11.6)  A.K-motif(12.82)  F.K-motif(13.75)  KL-motif(15.95)  K.L-motif(16) |
| F1NQL7  F1NEI9 | K...L-motif(11.6)  I.K-motif(13.06)  D.K-motif(13.66)  KL-motif(15.95)  K.L-motif(16) |
| Q05876  Q5ZKI1 | K...L-motif(11.6)  I.K-motif(13.06)  F.K-motif(13.75)  KL-motif(15.95)  K.L-motif(16) |
| Q5ZJQ4 | K...L-motif(11.6)  I.K-motif(13.06)  F.K-motif(13.75)  K.L-motif(16)  RD.K..N-motif(45.1) |
| Q5ZJ54 | L.K-motif(11.96)  V.K-motif(12.78)  A.K-motif(12.82)  I.K-motif(13.06)  K.L-motif(16) |
| Q90ZK6 | L.K-motif(11.96)  V.K-motif(12.78)  A.K-motif(12.82)  D.K-motif(13.66)  KL-motif(15.95) |
| Q5ZMB8 | L.K-motif(11.96)  V.K-motif(12.78)  A.K-motif(12.82)  D.K-motif(13.66)  K.L-motif(16) |
| E1BWF8  F1NN16 | L.K-motif(11.96)  V.K-motif(12.78)  A.K-motif(12.82)  F.K-motif(13.75)  K.L-motif(16) |
| F1NF28  E1BV83 | L.K-motif(11.96)  V.K-motif(12.78)  A.K-motif(12.82)  KL-motif(15.95)  K.L-motif(16) |
| Q5ZJL4  Q5ZMS3  Q5ZM91  F1NMC3  P09244 | L.K-motif(11.96)  V.K-motif(12.78)  I.K-motif(13.06)  D.K-motif(13.66)  KL-motif(15.95) |
| F1NLK6 | L.K-motif(11.96)  V.K-motif(12.78)  I.K-motif(13.06)  D.K-motif(13.66)  K.L-motif(16) |
| F1P3Q8 | L.K-motif(11.96)  V.K-motif(12.78)  I.K-motif(13.06)  F.K-motif(13.75)  KL-motif(15.95) |
| E1C1Y0 | L.K-motif(11.96)  V.K-motif(12.78)  D.K-motif(13.66)  KL-motif(15.95)  K.L-motif(16) |
| E1C0J4  E1BUS2 | L.K-motif(11.96)  V.K-motif(12.78)  F.K-motif(13.75)  KL-motif(15.95)  K.L-motif(16) |
| F1NZS9  F1NSA0 | L.K-motif(11.96)  A.K-motif(12.82)  I.K-motif(13.06)  D.K-motif(13.66)  F.K-motif(13.75) |
| Q5ZKC9 | L.K-motif(11.96)  A.K-motif(12.82)  I.K-motif(13.06)  D.K-motif(13.66)  KL-motif(15.95) |
| Q5F3X8 | L.K-motif(11.96)  A.K-motif(12.82)  I.K-motif(13.06)  F.K-motif(13.75)  KL-motif(15.95) |
| P56517 | L.K-motif(11.96)  A.K-motif(12.82)  I.K-motif(13.06)  F.K-motif(13.75)  K.L-motif(16) |
| Q5F3L1  F1NPW9  E1BSC0  Q5F424  E1BWJ5 | L.K-motif(11.96)  A.K-motif(12.82)  I.K-motif(13.06)  KL-motif(15.95)  K.L-motif(16) |
| Q5ZL80 | L.K-motif(11.96)  A.K-motif(12.82)  D.K-motif(13.66)  F.K-motif(13.75)  K.L-motif(16) |
| F1N8T3  Q5ZMT0 | L.K-motif(11.96)  A.K-motif(12.82)  D.K-motif(13.66)  KL-motif(15.95)  K.L-motif(16) |
| F1NC26  F1N9Y8  F1NV33 | L.K-motif(11.96)  A.K-motif(12.82)  F.K-motif(13.75)  KL-motif(15.95)  K.L-motif(16) |
| Q5ZHZ0 | L.K-motif(11.96)  I.K-motif(13.06)  D.K-motif(13.66)  F.K-motif(13.75)  K.L-motif(16) |
| Q6EE31 | L.K-motif(11.96)  I.K-motif(13.06)  F.K-motif(13.75)  KL-motif(15.95)  K.L-motif(16) |
| R4GIN8 | L.K-motif(11.96)  I.K-motif(13.06)  F.K-motif(13.75)  K.L-motif(16)  RD.K..N-motif(45.1) |
| Q9I9D1  Q5ZMQ9 | L.K-motif(11.96)  D.K-motif(13.66)  F.K-motif(13.75)  KL-motif(15.95)  K.L-motif(16) |
| Q9PU55 | V.K-motif(12.78)  A.K-motif(12.82)  I.K-motif(13.06)  D.K-motif(13.66)  KL-motif(15.95) |
| E1C3Q0 | V.K-motif(12.78)  A.K-motif(12.82)  I.K-motif(13.06)  F.K-motif(13.75)  K.L-motif(16) |
| H9L3L2  F1NVB1  F1NZJ2  E1BSW0 | V.K-motif(12.78)  A.K-motif(12.82)  F.K-motif(13.75)  KL-motif(15.95)  K.L-motif(16) |
| E1C1H4 | V.K-motif(12.78)  A.K-motif(12.82)  KL-motif(15.95)  K.L-motif(16)  RD.K..N-motif(45.1) |
| Q5ZIZ5 | V.K-motif(12.78)  I.K-motif(13.06)  D.K-motif(13.66)  F.K-motif(13.75)  KL-motif(15.95) |
| Q5ZLC7 | V.K-motif(12.78)  D.K-motif(13.66)  F.K-motif(13.75)  KL-motif(15.95)  K.L-motif(16) |
| Q7SZI5 | A.K-motif(12.82)  I.K-motif(13.06)  D.K-motif(13.66)  F.K-motif(13.75)  KL-motif(15.95) |
| Q5ZK01 | A.K-motif(12.82)  I.K-motif(13.06)  D.K-motif(13.66)  KL-motif(15.95)  K.L-motif(16) |
| E1C8Z7 | A.K-motif(12.82)  I.K-motif(13.06)  D.K-motif(13.66)  KL-motif(15.95)  RD.K..N-motif(45.1) |
| F1P555 | A.K-motif(12.82)  I.K-motif(13.06)  F.K-motif(13.75)  KL-motif(15.95)  K.L-motif(16) |
| E1C7R3  P53488 | I.K-motif(13.06)  D.K-motif(13.66)  F.K-motif(13.75)  KL-motif(15.95)  K.L-motif(16) |
| F1NJ10 | K...L-motif(11.6)  L.K-motif(11.96)  V.K-motif(12.78)  A.K-motif(12.82) |
| E1BZ62 | K...L-motif(11.6)  L.K-motif(11.96)  V.K-motif(12.78)  KL-motif(15.95) |
| F1NBA8  R9PXQ6  E1BZV7 | K...L-motif(11.6)  L.K-motif(11.96)  V.K-motif(12.78)  K.L-motif(16) |
| Q5ZIE6 | K...L-motif(11.6)  L.K-motif(11.96)  A.K-motif(12.82)  I.K-motif(13.06) |
| F1P0T0 | K...L-motif(11.6)  L.K-motif(11.96)  A.K-motif(12.82)  KL-motif(15.95) |
| F1N9C5  Q5ZJ38 | K...L-motif(11.6)  L.K-motif(11.96)  A.K-motif(12.82)  K.L-motif(16) |
| Q90679  Q5ZJZ2 | K...L-motif(11.6)  L.K-motif(11.96)  I.K-motif(13.06)  D.K-motif(13.66) |
| F1NX76 | K...L-motif(11.6)  L.K-motif(11.96)  D.K-motif(13.66)  K.L-motif(16) |
| Q6DV79 | K...L-motif(11.6)  L.K-motif(11.96)  F.K-motif(13.75)  K.L-motif(16) |
| F1NCH3  E1BU04  Q5ZKK5  E1BTP9 | K...L-motif(11.6)  L.K-motif(11.96)  KL-motif(15.95)  K.L-motif(16) |
| Q5F4C0 | K...L-motif(11.6)  V.K-motif(12.78)  A.K-motif(12.82)  KL-motif(15.95) |
| F1N9I5 | K...L-motif(11.6)  V.K-motif(12.78)  KL-motif(15.95)  K.L-motif(16) |
| F1NJL5 | K...L-motif(11.6)  V.K-motif(12.78)  K.L-motif(16)  RD.K..N-motif(45.1) |
| F1NI93  E1BYZ5 | K...L-motif(11.6)  A.K-motif(12.82)  I.K-motif(13.06)  K.L-motif(16) |
| Q5ZIG2  Q5ZHQ3 | K...L-motif(11.6)  A.K-motif(12.82)  D.K-motif(13.66)  KL-motif(15.95) |
| E1BX68 | K...L-motif(11.6)  A.K-motif(12.82)  D.K-motif(13.66)  K.L-motif(16) |
| F1NLG7  Q5ZJ21 | K...L-motif(11.6)  A.K-motif(12.82)  F.K-motif(13.75)  KL-motif(15.95) |
| F1NMM9  F1NG02  E1BU42 | K...L-motif(11.6)  A.K-motif(12.82)  KL-motif(15.95)  K.L-motif(16) |
| E1BZ00 | K...L-motif(11.6)  I.K-motif(13.06)  D.K-motif(13.66)  K.L-motif(16) |
| Q5ZJB4 | K...L-motif(11.6)  I.K-motif(13.06)  K.L-motif(16)  RD.K..N-motif(45.1) |
| F1NUY5  Q90732 | K...L-motif(11.6)  D.K-motif(13.66)  KL-motif(15.95)  K.L-motif(16) |
| Q5ZLN1 | L.K-motif(11.96)  V.K-motif(12.78)  A.K-motif(12.82)  I.K-motif(13.06) |
| E1C2P3  P00356  Q5ZJH9 | L.K-motif(11.96)  V.K-motif(12.78)  A.K-motif(12.82)  KL-motif(15.95) |
| P29332  F1NLT8  F1NH67  F1P5I1  E1BV45  F6SU35  E1C6E6 | L.K-motif(11.96)  V.K-motif(12.78)  A.K-motif(12.82)  K.L-motif(16) |
| F1P3Z0  E1BTJ9 | L.K-motif(11.96)  V.K-motif(12.78)  I.K-motif(13.06)  KL-motif(15.95) |
| F1NRZ6 | L.K-motif(11.96)  V.K-motif(12.78)  I.K-motif(13.06)  K.L-motif(16) |
| E1C4E1 | L.K-motif(11.96)  V.K-motif(12.78)  D.K-motif(13.66)  KL-motif(15.95) |
| P18652 | L.K-motif(11.96)  V.K-motif(12.78)  D.K-motif(13.66)  K.L-motif(16) |
| F1NRW5 | L.K-motif(11.96)  V.K-motif(12.78)  F.K-motif(13.75)  K.L-motif(16) |
| E1C0F5  E1BSS3 | L.K-motif(11.96)  V.K-motif(12.78)  KL-motif(15.95)  K.L-motif(16) |
| E1C0Z0  F1NCX7 | L.K-motif(11.96)  A.K-motif(12.82)  I.K-motif(13.06)  KL-motif(15.95) |
| Q5F3W0 | L.K-motif(11.96)  A.K-motif(12.82)  D.K-motif(13.66)  K.L-motif(16) |
| E1BX85 | L.K-motif(11.96)  A.K-motif(12.82)  F.K-motif(13.75)  KL-motif(15.95) |
| E1BZ78  F1NIM4  P47826  Q5ZMA2 | L.K-motif(11.96)  A.K-motif(12.82)  F.K-motif(13.75)  K.L-motif(16) |
| E1C2G3  Q5ZM62  Q7SX63 | L.K-motif(11.96)  A.K-motif(12.82)  KL-motif(15.95)  K.L-motif(16) |
| F1NLX2  Q9I8D6  F1NIP5 | L.K-motif(11.96)  I.K-motif(13.06)  D.K-motif(13.66)  KL-motif(15.95) |
| Q646T7  F2Z4L4 | L.K-motif(11.96)  I.K-motif(13.06)  D.K-motif(13.66)  K.L-motif(16) |
| E1BZJ3  F1N843 | L.K-motif(11.96)  I.K-motif(13.06)  F.K-motif(13.75)  KL-motif(15.95) |
| Q5ZJZ6  Q5ZMD2  Q5ZKA0  Q90647  Q5ZIL9  P84169  E1BYL7  Q5ZL97 | L.K-motif(11.96)  I.K-motif(13.06)  KL-motif(15.95)  K.L-motif(16) |
| F1NUQ0 | L.K-motif(11.96)  D.K-motif(13.66)  F.K-motif(13.75)  K.L-motif(16) |
| F1NWE5 | L.K-motif(11.96)  D.K-motif(13.66)  KL-motif(15.95)  K.L-motif(16) |
| F1NH72  Q5ZIF0  Q90955  F1NU56 | L.K-motif(11.96)  F.K-motif(13.75)  KL-motif(15.95)  K.L-motif(16) |
| Q5ZMN2 | V.K-motif(12.78)  A.K-motif(12.82)  I.K-motif(13.06)  F.K-motif(13.75) |
| Q5ZLN5 | V.K-motif(12.78)  A.K-motif(12.82)  D.K-motif(13.66)  KL-motif(15.95) |
| E1C6Q1 | V.K-motif(12.78)  A.K-motif(12.82)  D.K-motif(13.66)  K.L-motif(16) |
| F1NU17  F1NSY0 | V.K-motif(12.78)  A.K-motif(12.82)  F.K-motif(13.75)  KL-motif(15.95) |
| P84173  F1NGQ3 | V.K-motif(12.78)  I.K-motif(13.06)  D.K-motif(13.66)  K.L-motif(16) |
| Q90835 | V.K-motif(12.78)  I.K-motif(13.06)  F.K-motif(13.75)  K.L-motif(16) |
| Q9DEA3  F1P541 | V.K-motif(12.78)  I.K-motif(13.06)  KL-motif(15.95)  K.L-motif(16) |
| P05625 | V.K-motif(12.78)  I.K-motif(13.06)  KL-motif(15.95)  RD.K..N-motif(45.1) |
| E1BZE6  Q07598  Q5ZML3  Q5F4B9 | V.K-motif(12.78)  D.K-motif(13.66)  F.K-motif(13.75)  KL-motif(15.95) |
| F1NZI1 | V.K-motif(12.78)  D.K-motif(13.66)  F.K-motif(13.75)  K.L-motif(16) |
| Q5ZL57  E1BXG9 | V.K-motif(12.78)  D.K-motif(13.66)  KL-motif(15.95)  K.L-motif(16) |
| P42683 | V.K-motif(12.78)  F.K-motif(13.75)  KL-motif(15.95)  K.L-motif(16) |
| P38024 | A.K-motif(12.82)  I.K-motif(13.06)  D.K-motif(13.66)  F.K-motif(13.75) |
| E1BSZ6 | A.K-motif(12.82)  I.K-motif(13.06)  F.K-motif(13.75)  K.L-motif(16) |
| O42484 | A.K-motif(12.82)  D.K-motif(13.66)  F.K-motif(13.75)  KL-motif(15.95) |
| F1NYM4  F1N9B7 | A.K-motif(12.82)  D.K-motif(13.66)  KL-motif(15.95)  K.L-motif(16) |
| R4GFH1  F1NII4 | A.K-motif(12.82)  F.K-motif(13.75)  KL-motif(15.95)  K.L-motif(16) |
| F1N8Z4 | I.K-motif(13.06)  D.K-motif(13.66)  KL-motif(15.95)  K.L-motif(16) |
| Q04678 | D.K-motif(13.66)  F.K-motif(13.75)  KL-motif(15.95)  K.L-motif(16) |
| F1P0L5  P51913  F1P4E5  Q5ZLW0  E1BRU6 | K...L-motif(11.6)  L.K-motif(11.96)  V.K-motif(12.78) |
| Q5ZLF0 | K...L-motif(11.6)  L.K-motif(11.96)  A.K-motif(12.82) |
| E1C4W1  Q5ZIY4 | K...L-motif(11.6)  L.K-motif(11.96)  I.K-motif(13.06) |
| E1BQF9 | K...L-motif(11.6)  L.K-motif(11.96)  F.K-motif(13.75) |
| E1BTR2  E1C485  F1NME3 | K...L-motif(11.6)  L.K-motif(11.96)  KL-motif(15.95) |
| F1NIJ6  E1C4Q0  E1BY36 | K...L-motif(11.6)  L.K-motif(11.96)  K.L-motif(16) |
| R4GJ54 | K...L-motif(11.6)  V.K-motif(12.78)  A.K-motif(12.82) |
| R4GJF3 | K...L-motif(11.6)  V.K-motif(12.78)  F.K-motif(13.75) |
| F1NZA1 | K...L-motif(11.6)  V.K-motif(12.78)  KL-motif(15.95) |
| E1BVP1  Q5F3W5  Q5F418 | K...L-motif(11.6)  V.K-motif(12.78)  K.L-motif(16) |
| Q5ZJ64 | K...L-motif(11.6)  A.K-motif(12.82)  D.K-motif(13.66) |
| E1C2B1 | K...L-motif(11.6)  A.K-motif(12.82)  F.K-motif(13.75) |
| P26990  Q5ZMC1  E1BVN5  Q03669  F1NIW6 | K...L-motif(11.6)  A.K-motif(12.82)  KL-motif(15.95) |
| P51901  F1NIT0 | K...L-motif(11.6)  A.K-motif(12.82)  K.L-motif(16) |
| E1C8L5 | K...L-motif(11.6)  I.K-motif(13.06)  KL-motif(15.95) |
| Q5ZMK2  A5HUJ4  E1BYJ2  Q5ZJQ6  P79781 | K...L-motif(11.6)  I.K-motif(13.06)  K.L-motif(16) |
| F1NRC2  R4GGT7 | K...L-motif(11.6)  D.K-motif(13.66)  F.K-motif(13.75) |
| E1BYQ3 | K...L-motif(11.6)  D.K-motif(13.66)  KL-motif(15.95) |
| E1BYZ9  F1NFB8 | K...L-motif(11.6)  D.K-motif(13.66)  K.L-motif(16) |
| E1C8A2 | K...L-motif(11.6)  F.K-motif(13.75)  KL-motif(15.95) |
| E1BYV2  E1C3M4  E1BZS2  P42558  F1N9U0 | K...L-motif(11.6)  F.K-motif(13.75)  K.L-motif(16) |
| Q5ZII1 | K...L-motif(11.6)  KL-motif(15.95)  K.L-motif(16) |
| Q5ZLZ6  F1NCQ1 | L.K-motif(11.96)  V.K-motif(12.78)  A.K-motif(12.82) |
| Q90WD0  E1BXR0  Q5ZIV9 | L.K-motif(11.96)  V.K-motif(12.78)  F.K-motif(13.75) |
| F1NZU2  Q5F4C5  E1BV75 | L.K-motif(11.96)  V.K-motif(12.78)  KL-motif(15.95) |
| Q9DF37  E1BY08  F1NAQ8  F1NYM7 | L.K-motif(11.96)  V.K-motif(12.78)  K.L-motif(16) |
| F1NEX8 | L.K-motif(11.96)  A.K-motif(12.82)  I.K-motif(13.06) |
| O57535  F1NXP2 | L.K-motif(11.96)  A.K-motif(12.82)  D.K-motif(13.66) |
| Q5ZKZ8 | L.K-motif(11.96)  A.K-motif(12.82)  KL-motif(15.95) |
| F1NL52  Q5ZLA6  O42410  Q5ZLH1  F1NKL4 | L.K-motif(11.96)  A.K-motif(12.82)  K.L-motif(16) |
| E1BQG4 | L.K-motif(11.96)  A.K-motif(12.82)  RD.K..N-motif(45.1) |
| E1C2C2  E1BYG6 | L.K-motif(11.96)  I.K-motif(13.06)  D.K-motif(13.66) |
| Q5ZKP6  E1BVC8 | L.K-motif(11.96)  I.K-motif(13.06)  F.K-motif(13.75) |
| E1C600  F1NJ69  P01109 | L.K-motif(11.96)  I.K-motif(13.06)  KL-motif(15.95) |
| F1NS91 | L.K-motif(11.96)  I.K-motif(13.06)  K.L-motif(16) |
| E1BRA3 | L.K-motif(11.96)  I.K-motif(13.06)  RD.K..N-motif(45.1) |
| P62207 | L.K-motif(11.96)  D.K-motif(13.66)  F.K-motif(13.75) |
| F1NYI3  R4GF75  O42130 | L.K-motif(11.96)  D.K-motif(13.66)  KL-motif(15.95) |
| F1NH79  Q5ZM34  E1C7K1 | L.K-motif(11.96)  D.K-motif(13.66)  K.L-motif(16) |
| E1BYS6  F1NCE1  E1BT18  H9L3J8 | L.K-motif(11.96)  F.K-motif(13.75)  KL-motif(15.95) |
| C7G537 | L.K-motif(11.96)  F.K-motif(13.75)  K.L-motif(16) |
| E1C7U7  Q5F337  E1BQE4  Q5ZJ61  O93327  Q5ZMN4  F1NHL2  P47838  Q5ZJA5  Q5ZM13  Q6ITC7  Q91048  F1NPQ0  E1C6U4  Q5F486  R4GLK3  Q5ZKN1 | L.K-motif(11.96)  KL-motif(15.95)  K.L-motif(16) |
| Q8AYC9 | L.K-motif(11.96)  KL-motif(15.95)  RD.K..N-motif(45.1) |
| F1NPD3  Q805B1 | V.K-motif(12.78)  A.K-motif(12.82)  I.K-motif(13.06) |
| Q5ZLE8 | V.K-motif(12.78)  A.K-motif(12.82)  F.K-motif(13.75) |
| P16039  F1N9L9  E1C093 | V.K-motif(12.78)  A.K-motif(12.82)  KL-motif(15.95) |
| P50890  Q5ZIA0  P08284 | V.K-motif(12.78)  A.K-motif(12.82)  K.L-motif(16) |
| Q5ZJ02  F1NH78  Q5ZHM8 | V.K-motif(12.78)  I.K-motif(13.06)  D.K-motif(13.66) |
| Q9PTI4 | V.K-motif(12.78)  I.K-motif(13.06)  F.K-motif(13.75) |
| P50594 | V.K-motif(12.78)  I.K-motif(13.06)  KL-motif(15.95) |
| F1NSJ2 | V.K-motif(12.78)  I.K-motif(13.06)  K.L-motif(16) |
| F1NIK8  R4GLV4 | V.K-motif(12.78)  D.K-motif(13.66)  K.L-motif(16) |
| F1P1G4  Q8QFQ8  P62758 | V.K-motif(12.78)  F.K-motif(13.75)  KL-motif(15.95) |
| Q5ZME1  F1P3R7  F1NJZ2  F1NPS0  F1NPA3  P81021  F1NU68  F1ND57  Q5ZLI2  F1NHX1  Q98TX3 | V.K-motif(12.78)  KL-motif(15.95)  K.L-motif(16) |
| E1C1A7 | V.K-motif(12.78)  KL-motif(15.95)  RD.K..N-motif(45.1) |
| Q6B842 | A.K-motif(12.82)  I.K-motif(13.06)  KL-motif(15.95) |
| Q5ZIZ6  Q5F3T7  E1BZN6  E1C2D3  Q5ZLE9 | A.K-motif(12.82)  I.K-motif(13.06)  K.L-motif(16) |
| F1NN68  Q5F3R6 | A.K-motif(12.82)  D.K-motif(13.66)  F.K-motif(13.75) |
| E1C4N0  F1NZD3  Q5ZLS7  F1N9Y5  E1BSH7 | A.K-motif(12.82)  D.K-motif(13.66)  K.L-motif(16) |
| F1P0V5  F1N9J3 | A.K-motif(12.82)  F.K-motif(13.75)  KL-motif(15.95) |
| F1P0W9  F1NLM0  E1C455  E1BVH9 | A.K-motif(12.82)  F.K-motif(13.75)  K.L-motif(16) |
| F1NBT0 | A.K-motif(12.82)  F.K-motif(13.75)  RD.K..N-motif(45.1) |
| F1P3U1  P23913  R4GJH6  F1NGS7  E1C2A1  F1NZH3  P15771 | A.K-motif(12.82)  KL-motif(15.95)  K.L-motif(16) |
| E1BZ86 | A.K-motif(12.82)  K.L-motif(16)  RD.K..N-motif(45.1) |
| P09572 | I.K-motif(13.06)  D.K-motif(13.66)  KL-motif(15.95) |
| P08636  F2Z4K7  E1BVU8  F1P243  F1NEG1 | I.K-motif(13.06)  D.K-motif(13.66)  K.L-motif(16) |
| E1C8N4  P13474 | I.K-motif(13.06)  F.K-motif(13.75)  KL-motif(15.95) |
| Q5F3U4 | I.K-motif(13.06)  F.K-motif(13.75)  K.L-motif(16) |
| F1P0P4  E1BVR7  Q8UVD9 | I.K-motif(13.06)  KL-motif(15.95)  K.L-motif(16) |
| R4GI14  E1C1R4 | D.K-motif(13.66)  F.K-motif(13.75)  K.L-motif(16) |
| E1C6T8  Q5ZJW4  F1NYZ9  Q5ZLK1  Q5ZM72  Q5ZMC9  Q98TX7 | D.K-motif(13.66)  KL-motif(15.95)  K.L-motif(16) |
| E1C581 | D.K-motif(13.66)  KL-motif(15.95)  RD.K..N-motif(45.1) |
| E1C350  Q5ZHM9  R4GH86  Q8JHU1  E1BZL0  P40618 | F.K-motif(13.75)  KL-motif(15.95)  K.L-motif(16) |
| F1P5T2 | KL-motif(15.95)  K.L-motif(16)  RD.K..N-motif(45.1) |
| Q90ZH5  A3R064  F1P3V4  F1NRR5  E1BRU0  P79769  F1NB64  F1NDN4  E1C008 | K...L-motif(11.6)  L.K-motif(11.96) |
| Q5ZIR9  Q5ZJ03  E1C6Q5  Q5F465  E1BSI0 | K...L-motif(11.6)  V.K-motif(12.78) |
| H9KZF8  Q6VN51  E1C1N7  Q5ZM25  E1BQU5  F1NFJ5 | K...L-motif(11.6)  A.K-motif(12.82) |
| Q800L3  Q5ZKR7  Q5ZJF1 | K...L-motif(11.6)  I.K-motif(13.06) |
| P18359  Q98SN3  P84229  F1NN26 | K...L-motif(11.6)  D.K-motif(13.66) |
| P30352 | K...L-motif(11.6)  F.K-motif(13.75) |
| E1C9E9  E1BYM1  P20136  Q5ZLA7 | K...L-motif(11.6)  KL-motif(15.95) |
| E1BSG5  F1P521  P0CB05  F1P257  Q5XXA9  E1BTY1  F1NPV8  R4GFT6  Q9YH18  Q5ZLG1 | K...L-motif(11.6)  K.L-motif(16) |
| Q9PSW9  Q5F3C1  F1P4A5  F1NV18  F1N869 | L.K-motif(11.96)  V.K-motif(12.78) |
| Q5F452  D5M8S2  F1P331  E1BTL6  Q5ZKN2  H9L332 | L.K-motif(11.96)  A.K-motif(12.82) |
| F1NSW3  F1NW40  F1NQ19  Q5ZM44  Q5ZI25 | L.K-motif(11.96)  I.K-motif(13.06) |
| E1BW83  R4GIU0  F1P1X0  Q5F433  E1BWH5  E1C2F2  F1NYM1 | L.K-motif(11.96)  D.K-motif(13.66) |
| Q90972  F1P371 | L.K-motif(11.96)  F.K-motif(13.75) |
| R4GK17  A7E3K8  E1C717  E1C822  F1NB02  Q5ZJM9  F1NCI0  F1NQU3  E1BV65  R4GFE5  E1BZC4  F1NW29  H9KZT7  F1P5K0 | L.K-motif(11.96)  KL-motif(15.95) |
| E1BTA0  F1NNT7  Q5F3X4  R4GGJ0  F1NNE7  Q7T048  F1NGM0  F1N9S2  E1BVE1  Q90Z16  E1BW06  Q5ZL98  Q90660  O93481  F1NGJ4  E1BUM0  F1NJ73  P70082  Q8QG58  Q5ZKE7  E1BYP1  E1BWP4  F1P4M9  P27003 | L.K-motif(11.96)  K.L-motif(16) |
| Q5XKY5  Q5ZLX2  F1NVD2  F1NCI5  P09645  D5LPR1  Q5ZMK5 | V.K-motif(12.78)  A.K-motif(12.82) |
| E1C818  E1C7U8 | V.K-motif(12.78)  I.K-motif(13.06) |
| E1BVU1  R4GIF2 | V.K-motif(12.78)  D.K-motif(13.66) |
| Q5ZMN1  Q5ZI19  P54939  Q5F3Y0  Q5F470  Q5ZIN4  P00337 | V.K-motif(12.78)  KL-motif(15.95) |
| F1P1R3  E1C2H4  F1NY62 | V.K-motif(12.78)  K.L-motif(16) |
| P70081  F1NIQ9  F1NPL9  F1NWM5  Q5ZLA9  Q5ZLL6  F1NW97 | A.K-motif(12.82)  I.K-motif(13.06) |
| F1NJH4  R4GKD1  Q5F3Z5 | A.K-motif(12.82)  D.K-motif(13.66) |
| F1NLV4  Q58NQ4 | A.K-motif(12.82)  F.K-motif(13.75) |
| F1NHZ7  Q5ZLE7  F1NA82  F1NS33  E1BYA8  E1C270 | A.K-motif(12.82)  KL-motif(15.95) |
| E1C548  R4GLV5  F1NXW3  Q5ZLM0  F1NTK1  Q5ZLR6  E1C2V9  F1NPG2  Q90694  Q5ZLA5 | A.K-motif(12.82)  K.L-motif(16) |
| E1BQV7 | A.K-motif(12.82)  RD.K..N-motif(45.1) |
| F1NFC6  F1P062  F1NA45  F1P3Y0  F1NCZ2 | I.K-motif(13.06)  D.K-motif(13.66) |
| E1BQH2  F7BWA2  E1C6G6  Q5ZKU1  F1NLM1  F1NG47  R4GLV7  Q5ZJ22  P41125  Q5ZJT7  Q5ZL74 | I.K-motif(13.06)  KL-motif(15.95) |
| Q5ZK88  Q5ZM76  F1NJS6  Q5ZKD7  F1NSI8  E1C4Y2  F1P1K3  F1P2A4 | I.K-motif(13.06)  K.L-motif(16) |
| O13270  E1C122  F1NYA9  Q5F334  Q5ZID0 | D.K-motif(13.66)  KL-motif(15.95) |
| F1N8D4  E1BSM2  E1BV47  Q5F3U9  Q4W5Z4  F1NCX2  F1NR71  F1P1P3 | D.K-motif(13.66)  K.L-motif(16) |
| F1NJH1  P01994  E1BQ54  F1NBY5  P14315  E1BXC2 | F.K-motif(13.75)  KL-motif(15.95) |
| F1NG51  Q5ZM45  P62149  E1C0E2  F1P1N3  Q5ZIJ8  Q9DGG6  Q08200  F1NE58 | F.K-motif(13.75)  K.L-motif(16) |
| Q5ZIY8  E1C8M4  P05094  Q9YH06  E1BS88  E1BVF8  F1P4I3  P02112  F1NAK4  Q5ZMM3  F1P1G7  F1P556  Q5ZID6  Q5F4A3  E1C7J9  H9L0E7  Q5ZJF4  Q9PU45  F1NUK8  F1NBJ2 | KL-motif(15.95)  K.L-motif(16) |
| F1NKU2 | KL-motif(15.95)  RD.K..N-motif(45.1) |
| E1BX69 | K.L-motif(16)  RD.K..N-motif(45.1) |
| E1BVB0  F1N8Y3  Q8JHX2  F1NN84  F1NI39  Q5ZHS0  P08251  E1BXT5  F1NHM8  F1NJT9  F1NC47  E1C1H6  Q90999  O73777  F1NXR8  F1NW24  E1C959  F1P0R1  F1NT98  Q5ZJH5  P80566  F1P0S2  F1NNA2  F1NHV6  Q90683  F1NB52  E1BUH7  F1N903  E1C4E2  F1NPT0  E1BVB4  E1BQZ4 | K...L-motif(11.6) |
| F1NDV3  F1NEQ6  P48463  F1NPA2  P05081  P10360  F1P4D5  R4GM98  E1BXF3  Q5ZII6  P67883  Q5ZIJ9  F1NCE3  F1NAY8  P49416  F1NKQ0  Q5ZJA1  E1C541  E1BX13  Q5ZL34  R4GJP9  P22451  P58268  P01875  Q92071  Q5ZMW4  F1NKY6  R4GFB4  Q6U7I1  Q06A37  F1NB67  F1NMT9  E1BXY8  Q8JGM8  F1NCG7  E1BR04  F1NUM7  Q5ZM88  P61355  E1BWR2  F1NE09  O93277 | L.K-motif(11.96) |
| F1NE72  F1NFV8  E1C619  E1BRV1  D2D3P4  F1NG54  E1BXG0  Q2VB19  F1NH66  E1C4C7  F1NA68  E1BVP9  Q5ZKB1  F1NSI7  F1ND58  F1NH60  F1NC49  F1NZP1  Q5ZJL8  F1NEP9  F1NF89  E1BWT2  R4GJK4  E1C502  F1NEY5  F1NSA8  P24367  Q91955  F1N9R7  Q5F468  F1P226  E1BW57 | V.K-motif(12.78) |
| Q5ZKY0  E1C059  Q5F3A1  Q5F397  E1BYR9  Q5ZJJ9  F1NLK4  F1N8E6  R4GK64  F1NDZ9  Q08705  R4GMI7  F1P4G0  Q98SE2  Q9W7I5  A5HUM0  Q5F3R8  Q5ZKV8  F1NUU7  E1BWM5  F1P1E9  Q5ZMV9  E1BTM3  Q7T2T1  Q5ZIM6  F1P4X6  F1NFX3  F1NZ67  F1P0S9  F1NZC9  Q06066  E1C832  F1NKC1  E1C279  F1NM67  P08629  F1NZC4  F1NLK0  P08267 | A.K-motif(12.82) |
| F1P3P3  Q5F3T2  Q5ZMP6  F1NPI6  Q5ZKF5  Q5XNV3  Q5F3S6  Q5ZJH6  F1NVZ1  Q9W744  E1BSZ0  Q5F3J5  P07228  R4GF71  F1NDV9  E1BT75  E1C067  Q5F356  E1BYW9  P47832  E1BU31  F1NF80  Q5ZHW3  F1NYW0  Q5ZKF6  R4GGF3  P56519  F1NPM7  R4GFE1  F1NRI4  Q5F491  Q98TH5  P43347 | I.K-motif(13.06) |
| Q5ZI99  E1C3I1  F1NYB1  E1C8F7  Q5ZKK3  Q8QGH2  F1NZC1  E1BZT5  P32882  O93319  E1BRV6  P38531  E1BTD5  F1NSZ7  Q5F359  F1N8T0  R4GH53  F1NH33  E1C062  Q5ZMM7  F1NPJ9  E1BSD8  F1NYV4  R4GKR8  Q5ZI51  F1NKV0  E1C3P8 | D.K-motif(13.66) |
| E1C4J8  Q5ZKL7  E1C0P2  E1BZ63  E1C0E0  P42324  E1C626  F1NJL0  Q9PTR5  F1NYQ3  F1NQ24  F1P4J6  E1BWV9  O57389  Q9PV94  Q5ZI89  E1BQP3  E1BTV4  F1N8H4  A5HUM1  F1NT14  E1C4M9  F1NLF0 | F.K-motif(13.75) |
| F1NI66  F1P3C0  E1C7P7  P07630  Q5ZJ27  E1BUJ2  Q1T765  Q5ZKU4  P16236  Q5F3V3  F1NXN9  Q5F3G0  Q9W689  F1NL71  F1P4X5  Q5ZMR9  Q5ZK19  F1NL90  Q5ZL26  E1BUG9  E1BWG1  Q5ZLK9  F1P592  Q789A6  E1C129  Q5ZLX4  F1NFZ8  Q5ZLJ8  Q5ZJA9  H9L060  F1NFH6  F1P3C8  F1P0D2  F1NKE8  E1C0C3  P63247  F1NQ66  F1NV94  Q5ZIH1  E1BWQ3  E1C8K9  F1NTC8  F1P337  F6TLI2  E1BS06  F1P550  F1P1V1  E1C7V7  F1P5D8  Q5ZJG3  F1NJ25  E1BW98  P18870  R4GJX0  Q5ZLS3  R4GHV2  F1NBL4  P32429 | KL-motif(15.95) |
| F1NW50  F1NL19  F1NY51  R4GMK6  Q5ZKJ2  P02001  E1BZK0  E1BXG7  F1P569  F1N988  F1NYH8  F1NI89  F1N9P5  Q5ZI87  Q5ZKY5  Q5ZMG1  Q5F3M0  F1P0Z7  F1N862  F1NBV7  F1NU16  E1BY49  F1NCV7  E1C5R5  P02542  E1C189  F1N847  R4GI85  Q5ZM86  H9L0M7  E1BQW1  P02607  Q5ZI72  F1NKW9  F1NWU6  F1NV65  F1NE93  Q5ZIQ1  E1C1W0  F1NBI4  R4GLX2  F1NJI6  Q8UWG7  F1P010  Q5ZMN0  E1BXZ1  E1BQE1  E1C0Q6  E1C4G6  F1NII6  F1N8G9  F1NN74  Q9PTG6  E1C9D4  E1BX22  F1NLG1  Q5ZKG5  R4GFY3  E1BWS4  E1C878  F1P3I1  F1NDM6  Q9YH14  F1NJR5  Q5ZLY3  E1BX21  F1NED1  E1BTF7  F1P2S9  E1BXR3  F1NRK3  F1NY37  E1C3D8  Q5ZK87  Q5ZLS2  F1P3I0  E1BX57  E3VW39  F1NID0  F1NP22  E1BYQ6  O93480  E1BS61  R4GGW9  F1NG10  F1NFA4  E1BVI6  Q5ZL23  F1P571  E1BV81  Q5ZM73  F1NW06  H9KZY3  P79996  P28497  F1NUD1  E1BW48  E1BTG1  E1C5W9  F1NG19 | K.L-motif(16) |
| E1BS97  Q9I9E0 | RD.K..N-motif(45.1) |

The numbers within parentheses are the motif score.
